# Performance of National Maps of Watershed Integrity at Watershed Scales

**DOI:** 10.3390/w10050604

**Published:** 2018-05-05

**Authors:** Anne Kuhn, Scott G. Leibowitz, Zachary C. Johnson, Jiajia Lin, Jordan A. Massie, Jeffrey W. Hollister, Joseph L. Ebersole, James L. Lake, Jonathan R. Serbst, Jennifer James, Micah G. Bennett, J. Renée Brooks, Christopher T. Nietch, Nathan J. Smucker, Joseph E. Flotemersch, Laurie C. Alexander, Jana E. Compton

**Affiliations:** 1 U.S. Environmental Protection Agency, National Health and Environmental Effects Research Laboratory, Atlantic Ecology Division, Narragansett, RI 02882, USA; 2 U.S. Environmental Protection Agency, National Health and Environmental Effects Research Laboratory, Western Ecology Division, Corvallis, OR 97333, USA; 3 U.S. Environmental Protection Agency, Oak Ridge Institute for Science and Education (ORISE), National Health and Environmental Effects Research Laboratory, Western Ecology Division, 200 SW 35th St., Corvallis, OR 97333, USA; 4 School of Environmental and Forest Sciences, University of Washington, Seattle, WA 98195, USA; 5 National Research Council Post-Doctoral Fellow, National Academy of Sciences, Washington, DC 20001, USA; 6 Department of Earth & Environment, Florida International University, Miami, FL 33199, USA; 7 U.S. Environmental Protection Agency, National Center for Environmental Assessment, Washington, DC 20001, USA; 8 U.S. Environmental Protection Agency, National Risk Management Research Laboratory, Cincinnati, OH 45268, USA; 9 U.S. Environmental Protection Agency, National Exposure Research Laboratory, Cincinnati, OH 45268, USA

**Keywords:** watershed and catchment integrity, sustainable watershed management, anthropogenic stressors, aquatic connectivity, StreamCat, water quality, nitrogen, wetlands, lakes, streams

## Abstract

Watershed integrity, the capacity of a watershed to support and maintain ecological processes essential to the sustainability of services provided to society, can be influenced by a range of landscape and in-stream factors. Ecological response data from four intensively monitored case study watersheds exhibiting a range of environmental conditions and landscape characteristics across the United States were used to evaluate the performance of a national level Index of Watershed Integrity (IWI) at regional and local watershed scales. Using Pearson’s correlation coefficient (*r*), and Spearman’s rank correlation coefficient (*r_s_*), response variables displayed highly significant relationships and were significantly correlated with IWI and ICI (Index of Catchment Integrity) values at all watersheds. Nitrogen concentration and flux-related watershed response metrics exhibited significantly strong negative correlations across case study watersheds, with absolute correlations (|*r*|) ranging from 0.48 to 0.97 for IWI values, and 0.31 to 0.96 for ICI values. Nitrogen-stable isotope ratios measured in chironomids and periphyton from streams and benthic organic matter from lake sediments also demonstrated strong negative correlations with IWI values, with |*r*| ranging from 0.47 to 0.92, and 0.35 to 0.89 for correlations with ICI values. This evaluation of the performance of national watershed and catchment integrity metrics and their strong relationship with site level responses provides weight-of-evidence support for their use in state, local and regionally focused applications.

## 1. Introduction

Watersheds provide a functional context for assessing and managing aquatic ecosystems, as the water and materials from the surrounding landscapes drain to rivers, lakes, wetlands, groundwater, and downstream estuaries [1,2], which provide a range of ecosystem services, natural capital, and benefits to society [3–5]. These ecosystem services and benefits include supporting services (e.g., soil formation, nutrients, and primary production), provisioning goods and services (e.g., food, water, wood, fiber and fuel), regulating services (e.g., climate regulation, flood regulation and water purification) and cultural services, such as recreation and spiritual activities [3,6]. Many of these services and benefits are intrinsically linked to the natural dynamic character of hydrological processes and the intra-connected system of surface water and groundwater within watersheds [2,7]. Watershed and ecosystem processes operate at a variety of spatial and temporal scales, with processes operating at larger spatial scales generally influencing processes operating at smaller scales [1]. Structurally, watersheds are hierarchically organized (spatially nested) systems comprised of landscapes contributing to and providing key functional processes that generate and maintain aquatic ecosystem characteristics, including stream channel habitat structure, organic matter inputs, riparian soils, biotic and abiotic elements all connected by the flow of water [1,2]. Watersheds are topographically delineated areas that are drained by stream systems-the total land area above that drains to a point on a stream network. Local catchments represent the portion of landscape where surface flow drains directly into a stream segment, excluding any upstream contributions, whereas watersheds are comprised of hydrologically connected catchments consisting of all upstream catchments.

To effectively manage aquatic resources using a holistic watershed perspective, managers, stakeholders, and decision-makers need assessment approaches that integrate and synthesize information related to the functional attributes affecting watershed condition. There have been several recent developments in assessing and evaluating ‘watershed health’ that unite holistic ecosystem approaches with fundamental concepts from the field of landscape ecology. These examine functional ecohydrological processes and aquatic and landscape connectivity while accounting for the hierarchical nature of these processes occurring at multiple spatiotemporal scales [2,8–13]. Worldwide, watersheds are recognized as providing an important functional context for managing not only aquatic ecosystems and water resources as a physical unit, but also as socio-political units for management planning and implementation [14]. Internationally, water related legislative processes vary by country and organization with most governance focused on water resources including scarcity, water quality and water sanitation. Multi-national organizations such as the United Nations Food and Agriculture Organization (FAO), the World Bank, the Organization for Economic Co-operation and Development (OECD), the Global Water Partnership, and the European Union Water Framework Directive (WFD) have developed legislation, guidance, and principles for water governance systems, linked with effective stakeholder engagement and integrated systems-based approaches. Many of these entities employ Integrated Water Resource Management (IWRM) and Adaptive Management (AM) paradigms and share a common goal of equitably delivering sufficient water of good quality, while maintaining and improving the ecological integrity of water bodies [15,16].

In a recent effort to integrate the assessment and management of aquatic ecosystems within watersheds, Flotemersch et al. [2] constructed an operational definition and approach for evaluating ‘watershed integrity’, which the authors describe as ‘the capacity of a watershed to support and maintain the full range of ecological processes and functions essential to the sustainability of biodiversity and of the watershed resources and services provided to society’. This definition of watershed integrity builds and expands upon a foundation of biological and ecological integrity studies that define integrity of an ecosystem within an environmental context that includes natural variation and disturbance regimes, as well as anthropogenic alterations and disturbances [17–19]. The ultimate factor affecting ecological integrity of aquatic ecosystems is human activity, and ecological integrity is inversely related to human impacts on ecosystems [20]. The major anthropogenic disturbances linked to the degradation of aquatic ecosystem integrity are associated with population growth, land use alterations, increases in impervious surfaces, agriculture, mining, oil and gas extraction, point and diffuse polluted runoff, riparian and instream channel modifications, water impoundment and extraction [21]. Within watersheds, river and stream ecosystems are exhibiting increasingly disturbed conditions associated with chemical pollution and physical habitat alterations, directly reducing the integrity and health of these aquatic ecosystems [22,23].

Focusing on the key functional elements and processes necessary to maintain watershed provision of services and the risk factors that degrade these functions, Flotemersch et al. [2] utilized a human health analogy to construct an operational definition for an Index of Watershed Integrity (IWI). Similar to how practitioners in the human health field assess health and fitness by screening for the presence of risk factors or indicators (e.g., high blood pressure, cholesterol levels, being overweight and inactive) associated with various illnesses, Flotemersch et al. [2] identify six key functions that watersheds provide and the associated risk factors or landscape stressors that have been shown to interfere with and degrade these functions (e.g., urban and agricultural land use, stream channelization, transportation infrastructure). The framework developed by Flotemersch et al. [2] was then used by Thornbrugh et al. [24] to develop a watershed integrity assessment for the conterminous U.S. (CONUS).

**Figure 1:**
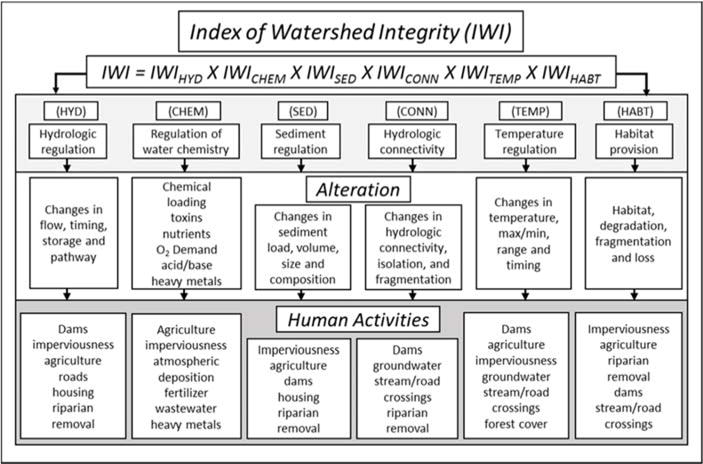
Conceptual model of the calculation of the Index of Watershed Integrity including human activities that produce stress and degrade key functions in watersheds. Source: Ecological Indicators as included in Mapping watershed integrity for the conterminous United States, Thornbrugh et al. [24].

The six key watershed functions described in detail in Flotemersch et al. [2] and Thornbrugh et al. [24] are: (1) hydrologic regulation (HYD), (2) regulation of water chemistry (CHEM), (3) sediment regulation (SED), (4) hydrologic connectivity (CONN), (5) temperature regulation (TEMP), and (6) habitat provision (HABT) (Figure 1 and Table 1). The integrity of each of these six key watershed functions is based on the relative presence of specific landscape stressors that affect them. The IWI is calculated by taking the product of these six values, because each of these functions is a critical component of watershed integrity and the functions are not substitutable [2].

Using the operational definition of watershed integrity provided by Flotemersch et al. [2] and landscape stressor data from StreamCat [13], Thornbrugh et al. [24] derived and mapped the IWI for 2.6 million stream segments in the National Hydrography Dataset Plus Version 2 (NHDPlusV2, Horizon Systems Corporation, Herndon, VA, USA) to visualize spatial patterns across the CONUS. This national map of IWI was developed using first order approximations of relationships between landscape stressors and the six watershed functional components [24] (Table 2, Table 3). A related Index of Catchment Integrity (ICI) was also developed using local drainages of individual stream segments, excluding upstream contributions. These non-nested catchments do not overlap and characterize the local influence of the catchment, not the watershed. It should be noted that the IWI and ICI only incorporate risk factor data for stressors having data layer coverage for the entire CONUS. Therefore, they do not include all the stressors that Flotemersch et al. [2] conceptually associated with the six watershed functions (Table 1, Figure 1).

Since ‘watershed integrity’ is a theoretical concept, and not physical property that can be measured, it is not possible to directly validate the performance of the IWI and ICI. However, Thornbrugh et al. [24] hypothesized that there is a relationship between these indices of integrity and aquatic ecosystem condition. In this study, we use a weight-of-evidence approach to quantitatively examine the strength of correlation between each of the indices of watershed and catchment integrity and site-level aquatic resource response metrics for streams, lakes and wetland resources representing watershed infrastructure. These site-level metrics were defined for four intensively monitored case study watersheds exhibiting a wide range of environmental conditions and landscape characteristics across the United States. We use the values for IWI and ICI derived by Thornbrugh et al. [24] for the national-scale mapping effort for stream segments in the four case study watersheds to compare with site-level metrics that are known indicators of aquatic ecosystem impairment and have been derived uniquely to characterize and/or study specific impacts within the focal systems. The goal was to evaluate how the IWI and ICI perform relative to other indicators of aquatic ecosystem health derived independently from local and intensive aquatic resource assessment investigations.

The objectives of this study were twofold: (1) to evaluate the performance of the IWI, ICI and their respective six associated functional components using local scale (i.e., site-level) metrics and response variables; and (2) to identify specific aquatic characteristics and indicator responses that are correlated with the national watershed integrity metrics, as well as data for specific local site-level characteristics that may need refinement to improve the validity and robustness of these national metrics. The IWI and ICI values derived by Thornbrugh et al. [24], along with the associated six functional watershed components, could provide a pragmatic tool for states, tribes, other federal agencies, and watershed councils for identifying and prioritizing protection of watersheds with high integrity as well as identifying critical components of watersheds that can be targeted for conservation and restoration. The correlations between the IWI/ICI and known indicators of aquatic ecosystem impacts derived for aquatic resources within specific watersheds should lend confidence to the utility of these metrics as indicators of a watershed’s integrity at any scale.

## 2. Materials and Methods

### 2.1. Study Areas

**Figure 2:**
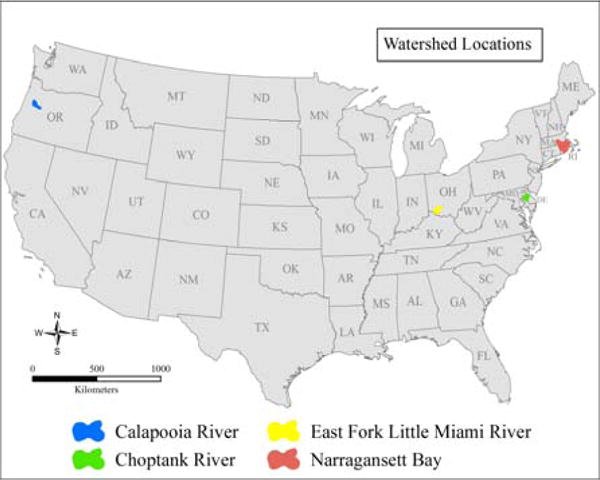
Locus map for location of four case study watersheds across the United States.

We selected four intensively monitored watersheds from across the United States having data from multiple sites throughout the watershed. The four case study watersheds range in size, elevation, landscape, and demographic characteristics (Table 4; Figure 2).

#### 2.1.1. Calapooia River Watershed

**Figure 3:**
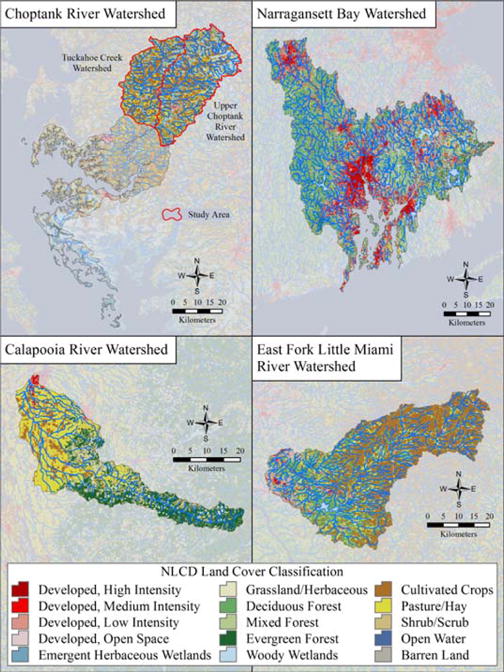
Landscape maps of land use and land cover characteristics for four case study watersheds using 2011 National Land Cover Data.

The Calapooia RiverWatershed (CRW) is part of theWillamette drainage and Columbia River basin within the Western Cascades mountain range in Oregon. The CRW encompasses an area of 945 km^2^ with elevation ranging from 1562 m at the headwaters and summit of Tidbits Mountain to less than 57 m at the confluence with the Willamette River. Land cover in the higher elevations is largely evergreen forest, with lower elevations more dominated by agricultural practices (Table 4). Developed areas occupy the smallest proportion of land use (5%) in the watershed and are concentrated in the most western portion of the watershed at lower elevations (Figure 3). While there have been significant land use alterations in the form of timber harvesting and water diversion for the agricultural production of grass seed farming, the Calapooia River corridor still contains large areas of intact riparian forests, backwater sloughs and large remnants of mudflat and vernal pool communities. A mixture of rain and snow contribute to the Calapooia River annual flow with winter precipitation as rain in the lower elevations of the watershed and snow in the mountainous areas above 1067 m. Summers are hot and dry with only 5% of the annual precipitation occurring between July and September [26].

#### 2.1.2. Choptank Study Area: Upper Choptank River and Tuckahoe Creek Sub-Basins

The Choptank River (CHOP) is a major tributary of the Chesapeake Bay that originates in Choptank Mills, Delaware and flows southwest through Maryland’s Eastern Shore. The entire watershed is located within the coastal plain of the Delmarva Peninsula in the Mid-Atlantic region. The Choptank was a U.S. Department of Agriculture Conservation Effects Assessment Project (CEAP) Benchmark watershed [27]. The Choptank study area encompasses 1070 km^2^ in the headwaters of the Upper Choptank and Tuckahoe Creek sub-basins within the northern portion of the Choptank River watershed (Figure 3). The major land uses within the Choptank study area are agriculture in the form of poultry farms, forestry, and large-scale corn, soybean, and small grains production. Built-up or urban land use accounts for about 6% of the study area region (Table 4). The Choptank study area is relatively flat with a maximum elevation of 36 m with wetlands comprising 20% of the watershed-study area. Most of these wetlands are characterized as forested, including wetland depressions (e.g., ‘Delmarva bays’: elliptical depressions surrounded by sandy upland) [28], wetland flats, and riparian wetlands. The study area is characterized by a humid, temperate climate with average annual precipitation of 111 cm occurring throughout the water year [29]. Approximately 50% of annual precipitation is lost to the atmosphere via evapotranspiration while the remainder recharges ground water or enters streams via surface flow [30].

#### 2.1.3. East Fork Little Miami River Watershed

The Little Miami River, located in southwestern Ohio in the Ohio River drainage basin, contains some of Ohio’s most scenic and diverse riverine habitat and is designated a State and National Scenic River by the Ohio Environmental Protection Agency: http://www.epa.state.oh.us/dsw/tmdl/LittleMiamiRiver.aspx. The case study area, the East Fork Little Miami River (EFLMR) watershed, is a major tributary of the Little Miami River watershed and encompasses an area of 1293 km^2^. Along its course the river drops from an elevation of 365 m to 149 m with an average gradient of 1.4 m/km. In 1978, the U.S. Army Corps of Engineers impounded a section of the East Fork Little Miami River by constructing an earthen dam at river km 33, creating an 874-hectare reservoir (Harsha Lake) stretching approximately 16 km upstream from the dam. Draining agricultural lands, Harsha Lake functions as both a significant sink and source of nitrogen [31]. The reservoir was primarily intended to provide flood control but is also used for recreation (boating and fishing) and is a source of drinking water for a 72 million liters per day treatment plant serving residents of Clermont County, Ohio [32]. The predominant land uses of the EFLMR watershed are rural and agricultural lands (55% including hay/pasture) found mostly in the headwaters and upper portions of the watershed. Forested lands are dispersed throughout the central parts of the watershed (32%); with developed and urban lands more concentrated in the lower portions (11%) (Figure 3, Table 4).

The EFLMR watershed has a temperate climate characterized by well-defined winter and summer seasons. The average annual total precipitation ranges from 104–109 cm, with about 40% falling during the growing season between May and August. The months with the least amount of precipitation are January, February, and October, all with average monthly totals of less than 7 cm [33].

#### 2.1.4. Narragansett Bay Watershed

Narragansett Bay is the largest estuary in New England and is located primarily in Rhode Island; however, 60% of the watershed contributing to the Bay is in Massachusetts. The Narragansett Bay Watershed (NBW) area covers 4421 km^2^ and is one of the most densely populated watersheds in the United States, with 442 persons/km^2^ [34] and roughly 1.9 million people residing within the watershed [35]. Three main rivers (the Blackstone, Taunton, and Pawtuxet Rivers) provide approximately 80% of the freshwater inputs to Narragansett Bay with an average of 7.9 billion liters per day of fresh water [35]. Roughly 39% of the watershed is forested and 35% is urban or developed land, with the highest percentages of urban land located adjacent to major waterways along the rivers draining directly to Narragansett Bay (Figure 3, Table 4). Saltwater and freshwater wetlands comprise about 15% of the watershed, while agricultural lands are a small portion of the watershed with only about six percent of the land in cultivated crops or hay/pasture. The geographic position of the Narragansett Bay watershed in the mid-latitudes and its coastal location places it near the polar jet stream, providing a climate of frequently changing weather from the regular passing of low pressure storms associated with the jet stream. The area experiences cold winter and warm summer air masses from the continental interior and the moderating and moistening influence of the western Atlantic Ocean, providing wide-ranging daily and annual temperatures. The annual average precipitation is about 125 cm (Table 4).

### 2.2. Case Study Response Variables

The four case studies discussed herein were independently conceived and executed from this study, and represent an array of aquatic ecosystem conditions, as well as temporal and spatial scales. Consequently, each study relied on a unique set of response variables and metrics. These site-level response metrics were derived independently to characterize and represent stressors and impacts within the focal watershed. Taken collectively, these differences present an opportunity to comprehensively evaluate how the watershed and catchment integrity indices perform across a range of conditions and settings typical of those encountered in the United States and abroad.

#### 2.2.1. Calapooia River Watershed

Eighteen watershed attribute response variables or metrics were developed in the CRW: nine nitrogen-related response variables, five response variables related to the physical habitat of streams, three stream temperature response variables, and a multimetric fish index (Table 5). Descriptions of all 18 response variables are provided in Table 5, and detailed methods for these 18 response variables are provided in Supplementary Materials. Sampling sites were chosen to capture broad gradients in physical setting, stream size, and watershed condition. Sampling sites were almost entirely located on privately owned residential, agricultural, or commercial forest lands which make up the vast majority of the stream network length, thus were severely constrained by landowner permissions (stream sample site locations in Figure S1). At each site sampled for fish assemblages, we collected water chemistry, temperature, and physical habitat information, unless landowners specifically requested that we abstain from doing so. The two dams in the basin were removed in 2008 and 2011. We did not sample within 1000 m above or below existing or former dam sites.

Nitrogen-related response variables included the monthly average and range in monthly average nitrate concentrations at 53 stream sampling sites [36,37], total nitrogen input, export and retention at the same 53 stream sampling sites from 2003–2006 and 2009–2011 [37,38] (see detailed methods in Supplementary Materials) and nitrogen-stable isotope ratios (^15^N/^14^N expressed as δ^15^N) measured from chironomids collected at 31 stream site locations within the CRW from 2013–2015 [38]; see detailed methods in Supplementary Materials.

Physical habitat data were collected from 2013–2015 for 20 streams within the CRW during the summer low-flow season. The following two stream physical habitat metrics were extracted for comparison and analysis to capture functional processes of interest that had been previously identified by Kaufmann [39] and Kaufmann et al. [40] (see Table 19 in [40]) Table 19 as among those most reliable and commonly used: sediment embeddedness (sedembed), a measure of the degree to which substrate cobbles and gravels are encompassed by finer sediments; and riparian vegetation cover (vegcovrip), an index of riparian vegetation density and complexity.

Stream water temperature was measured using temperature loggers (Optic TidBits model TBI32; Onset Computer Corp., Pocasset, MA, USA) placed in a well-mixed portion of the stream channel following methods of Dunham et al. [41]. The maximum summer temperature metric was derived from a database containing seven years (2009–2015) of 30-min time series temperature logger data from 87 established sites within the Calapooia basin. Maximum summer temperature was defined as the absolute maximum observation during the warmest period of the year in western Oregon, July–August (see detailed methods in Supplementary Materials). The maximum temperature value across all years considered was assigned to each site as a final representative maximum summer temperature metric. Amplitude and phase metrics for stream temperatures were calculated following the methods of Maheu et al. [42] to fit a sine curve to continuous time series data for each sample site and examine the magnitude and timing of temperature change throughout the year. This method provides a generalizable index of thermal regime magnitude (amplitude) and timing (phase). The index of thermal regime timing, phase, was calculated for 64 stream site locations within the CRW.

Fish sampling data used to derive multimetric indices (MMIs) in the Calapooia basin were collected between 2010 and 2014 using methodologies consistent with those used by US EPA’s Environmental Monitoring and Assessment Program (EMAP) [43] and the US EPA’s National Rivers and Streams Assessment (NRSA) [38]. In total, 50 sites were visited over four years (no sampling occurred in 2012) and ranged from forested headwater streams in the Cascade mountains to lowland reaches in high density agricultural areas of the Willamette Valley. Study reaches were 40 times the active channel width and were sampled using a single pass with backpack electrofishers [44]. Sampling occurred throughout the year but for consistency, only data collected during the NRSA index period (June through September) were used for this analysis (summer low-flow conditions). MMIs were calculated following methods set by Whittier et al. [45] (see detailed methods in Supplementary Materials).

#### 2.2.2. Choptank Study Area: Upper Choptank River and Tuckahoe Creek Sub-Basins

There have been numerous studies evaluating the various methods for characterizing and measuring the physical, biological, and chemical interactions and connectedness of wetlands and streams, principally for the determination of wetland jurisdiction and protection [29,46–50]. The connection between wetlands and streams can influence the structure, function, and environmental conditions of each via surface water and groundwater interactions and through the hyporheic zone, a subsurface area adjacent to the stream channel where stream and local ground waters mix [29].

For this study several hydrologic connectivity metrics (described below) were developed using GIS data layers to measure the interconnectivity of streams and wetlands within the Choptank Study area (Figure 3). By measuring the physical intersection of wetlands and streams within catchments we can use these metrics as a proxy of hydrologic connectivity to compare with the IWI/ICI values and their respective CONN (hydrologic connectivity) functional components. The National Wetlands Inventory Version 2 (NWI V2) from the US Fish and Wildlife Service (FWS) was used to obtain the wetlands spatial data layers of the National Spatial Data Infrastructure (NSDI) https://www.fws.gov/wetlands/data/Mapper.html.

Since riverine wetlands are connected to waterbodies by definition, these wetland polygons were removed (there were no estuarine or marine wetlands in the study area) from the NWI V2 GIS data layer and analysis, leaving only palustrine wetland polygons. Therefore, our analyses focus on quantifying ‘non-riverine’ wetland and stream connectivity at the catchment scale. Two different stream network representations were used to characterize the Choptank study area to evaluate if this affected connectivity estimates and subsequent correlations with the IWI/ICI values: the 1:100,000-scale NHDPlusV2; and the semi-automated high resolution Light Detection and Ranging (LiDAR) derived stream network developed by Lang et al. [30].

Based on numerous wetland connectivity studies and a body of literature [46,51–54] we applied a set 10-m geospatial buffer distance from the edge of the stream network using ArcGIS (version, ArcGIS Version 10.3; Esri, Redlands, CA, USA) to account for the spatial error in the NHDPlusV2 stream channels (defined using NHD Flowline features) [55]. The ArcGIS ‘buffering tool’ [56] buffered the streams laterally from each side of the stream channel and extended the 10-m buffer from the beginning or end of the stream channel. Using the ArcGIS ‘tabulate by intersection tool’ the non-riverine NWI V2 wetland polygons (excluding tidal or riverine types) that intersected the 10-m buffered NHDPlusV2 stream channels in the Choptank study area were extracted to develop independent measures of intersecting wetland-stream connectivity (aquatic connectivity) to be compared with the IWI/ICI values. The same GIS stream buffering and ‘tabulate by intersection tool’ GIS methods described above were repeated with the higher resolution semi-automated LiDAR derived stream network data layers. The intersecting non-riverine wetland-stream connectivity using both stream networks was estimated by evaluating the following five metrics for wetlands at the catchment scale: the count of wetland polygons intersecting stream channels in a catchment; the count of whole wetland polygons within a catchment (i.e., wetland polygon is contained completely within catchment); count of partial wetland polygons in a catchment (i.e., portion of the wetland polygon spreads across catchment boundary); the percentage of areal coverage for wetland polygons intersecting stream channels in a catchment; and the area of wetland polygons intersecting stream channels in a catchment. All five of these aquatic connectivity metrics were used as response variables to compare with the IWI/ICI values and six associated functional components (Table 5).

#### 2.2.3. East Fork Little Miami River Watershed

Five nitrogen metrics (total-N, total ammonium, total nitrate/nitrite, inorganic-N, and fraction of total-N as inorganic-N) were calculated using two different summary metrics (annual mean and annual range), for a total of 10 nitrogen-related response variables (Table 5). The data were developed using intermittently collected surface water chemistry data from 85 stream site locations in the EFLMR watershed from 2005–2015. Nitrogen species were analyzed using a flow injection auto analyzer (QuickChem, Lachat Instruments, Loveland, CO, USA) and the manufacturer’s methods for total nitrate [57], total ammonium nitrogen [58] and total nitrogen [59]. Data were organized by month between 2005–2015 and monthly average values were calculated for each site-analysis pairing (see Supplementary Materials for more detailed methods). However, the number of years of data varied for each EFLMR site. Monthly averages for site-analysis pairings with less than 3 observations for a given month were left blank. Annual averages were calculated by averaging the 12 monthly average values for each site-analysis pairing. Sites with data for less than 10 separate months were removed from further analysis. This left 44 sites that could be associated with IWI/ICI values for further analysis (stream sample site locations in Figure S2). Annual mean and range values were calculated using the monthly summaries (see Supplementary Materials for more detailed methods). Annual mean and range values were then log10 transformed. All five nitrogen metrics and their related summary metrics (annual mean and annual range) were used as response variables to compare with the IWI/ICI values and six associated functional components (Table 5).

#### 2.2.4. Narragansett Bay Watershed

##### Stream Chemistry

To examine landscape effects on stream water quality along a development gradient, a stratified, spatially balanced random sampling design [60] was used to select stream sampling sites along a gradient of impervious cover (IC) ranging from 1 to 48% within the NBW. A single grab sample for surface water chemistry was collected at 77 stream sites within the NBW between July and October in 2012 (stream sample site locations in Figure S3). Water samples were filtered through 0.45 μm pore size membranes and analyzed for PO_4_^3−^, NO_3_-N (as NO_3_+NO_2_−N), NH_4_^+^, dissolved organic carbon (DOC), SO_4_^2−^, Cl^−^, Ca^2+^, Mg^2+^, Na^+^, and K^+^, and unfiltered samples were used to measure total P and total N using US EPA approved protocols [61,62]. TN and NO_3_-N were measured using the cadmium reduction method and NH_4_+ was measured using the phenolate method. Of the 77 sampled stream sites, 71 sites were associated with IWI/ICI values; Cl^−^, TN, NO_3_-N and NH_4_^+^ were chosen as response variables reflecting watershed development impacts and anthropogenic sources of nitrogen in streams to relate to IWI/ICI and six associated functional component indices (Table 5).

##### Stable Isotope Ratios of *δ*^15^N and *δ*^13^C for Periphyton

Periphyton was simultaneously collected at the same 77 stream sites within NBW where single grab sample surface water chemistry was collected (described in section above) between July and October 2012 [60]. Stable isotope ratios of periphyton samples were determined using a continuous flow isotope ratio mass spectrometer (Isoprime 100 Mass Spectrometer, Elementar Americas, Mt. Laurel, NJ, USA) and reported as per mil differences (‰) between samples and reference materials (δ^15^N and δ^13^C) [62]. Of the 77 sampled stream sites, 69 sites were associated with IWI/ICI values, and the measured nitrogen-stable isotope ratio (^15^N/^14^N expressed as δ^15^N; see Supplementary Materials for more detailed methods) of periphyton was used as a response variable reflecting the effects of different nitrogen sources on stream networks [62] to compare with IWI/ICI values and six associated functional components (Table 5).

##### Stable Isotope Ratios of *δ*^15^N and *δ*^13^C for Benthic Organic Matter in Lakes

Increases in stable isotope ratios of δ^15^N and δ^13^C of benthic organic matter (BOM) collected from surficial sediments in lakes are associated with increases in impervious surface and population density and decreases in forested land in watershed and buffer zones surrounding lakes [63]. We used stable isotope ratios collected from BOM samples in lakes to evaluate how these measures of aquatic condition are correlated with IWI/ICI values. Samples of benthic organic matter were collected from the littoral zone of 51 lakes within the NBW using a hand-held piston coring sampler during the months between May through November from 2012–2013. Stable isotope ratios of these BOM samples were determined using the same methods described above for measuring stable isotope ratios of δ^15^N and δ^13^C in periphyton (see Supplementary Materials for detailed methods). Many of the lakes within the NBW are stream fed and hydrologically connected to the stream networks, enabling all 51 sites to be associated with IWI/ICI values using the NHDPlusV2 flowlines that flow through these lakes. The measured δ^15^N of BOM from these 51 lakes was used as a response variable to compare with the IWI/ICI values and their six associated functional components (Table 5).

### 2.3. Explanatory Predictor Variables

The IWI and ICI indices were developed using the US EPA’s StreamCat dataset [13], which was built on the NHDPlusV2 [55,64], a 1:100K national digital stream network containing more than 2.6 million stream segments in the CONUS. The StreamCat dataset links national landscape geospatial layers including human-related stressors (e.g., roads, dams, mines, imperviousness, etc.) to stream segments at the catchment and watershed levels as defined in Hill et al. [13]: catchment represents the portion of the landscape where surface flow drains directly into an NHD stream segment, excluding any upstream contributions; and watershed refers to the set of hydrologically connected catchments, consisting of all upstream catchments that contribute flow into any catchment.

Using the geographic location of each sample site within each case study watershed, we identified sites located on 1:100K NHDPlusV2 streams and associated the corresponding watershed/catchment IWI/ICI values for the target catchment using the unique ID or “COMID” (NHDPlusV2) for each stream segment that was sampled. To reduce spatial autocorrelation with nested sampling sites, in cases where there was more than one sampling site within a catchment the sampling site that was the furthest downstream within the catchment was used for the response variable value. For lake samples (sampled only in the Narragansett Bay watershed) we associated the IWI/ICI values for the NHDPlusV2 stream segment that runs through each lake waterbody with the corresponding sampled lake site.

Response variables from each study site were also examined for correlation with select individual landscape explanatory variables from StreamCat (i.e., % forest, % urban, % agriculture) using the unique COMID for each stream segment sampled. This was done because any effect observed with the IWI or ICI could be due to one of these dominant land use variables. In such a case, development of the IWI or ICI, which is complex and requires significant effort, would be unnecessary. Comparing correlations between response variables and both the integrity indices and the separate land use indicators allowed us to determine whether there was value added from the IWI and ICI. For each response variable and scale (watershed or catchment), we calculated the following: %Max, equal to the IWI or ICI absolute correlation value divided by the maximum absolute correlation value; Rank, which is the rank of the IWI or ICI absolute correlation value; and #Exceed, or the number of individual landscape variable absolute correlations that the IWI or ICI absolute correlation exceeded.

### 2.4. Statistical Analyses

We calculated Pearson’s correlation coefficient (*r*) to measure the strength of the linear relationship and association between the IWI/ICI values and the response variables within each watershed. The six functional components or indices associated with each of the IWI and ICI values were also compared to each of the response variables using Pearson’s correlation coefficient. One exception was for the Choptank Watershed study area; because the response variables developed within this watershed were not normally distributed, Spearman’s rank correlation coefficient (*r_s_*,) was used as a non-parametric rank statistic to measure the strength of the association between response variables and the IWI/ICI and six functional component indices.

We evaluated the strength of association and direction of the linear correlation of 29 response variables from across the four case study watersheds, with IWI, ICI and the six functional component indices of each (n = 14), for a total of 406 correlations. We characterize the strength of these correlations using five classification categories: ‘very strong’ (0.80–1.0); ‘strong’ (0.60–0.79); ‘moderate’ (0.40–0.59); ‘weak’ (0.20–0.39); ‘very weak’ (0.0–0.19). All correlation analyses were performed in R Software Version 3.4.1 [65]; all data and R packages used for developing figures and analyses are publicly available: https://github.com/usepa/watershed_integrity [66].

## 3. Results

### 3.1. Indices of Watershed and Catchment Integrity

**Figure 4:**
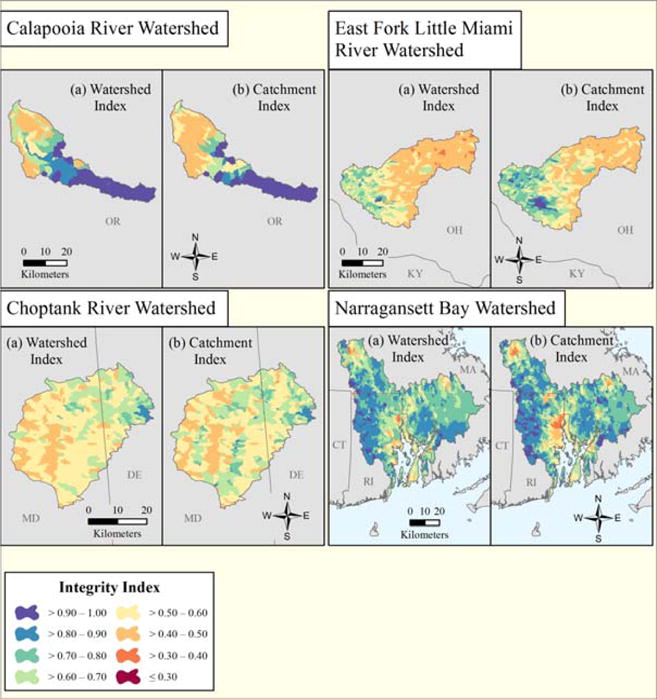
Watershed and catchment integrity maps for the Calapooia River, Choptank River, East Fork Little Miami River and Narragansett Bay watersheds. (**a**)Watershed index; (**b**) Catchment index for each watershed.

**Figure 5:**
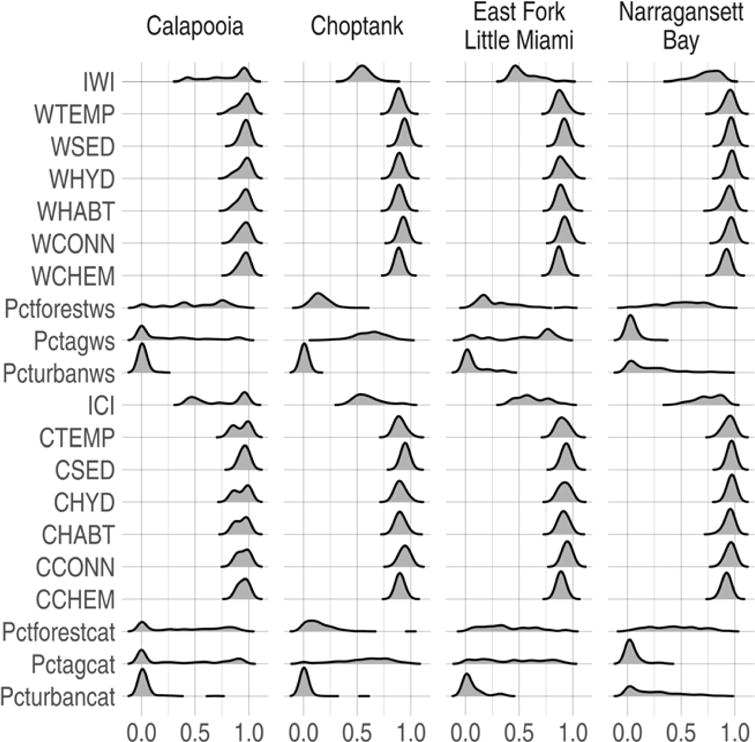
The distribution of Indices of Watershed (IWI) and Catchment Integrity (ICI) values, six functional component indices, and watershed land cover for sampled sites within each case study watershed. Functional components at watershed (w) and catchment (c) scales: HYD = hydrologic regulation; CHEM = regulation of water chemistry; SED = sediment regulation; CONN = hydrologic connectivity; TEMP = temperature regulation; and HABT = habitat provision. Land cover variables: % forest watershed/catchment (pctforestws/pctforestcat), % urban watershed/catchment (pcturbanws/pcturbancat), % agriculture watershed/catchment (pctagws/pctagcat).

Maps of the four case study watersheds show the entire range and spatial distribution of the indices of watershed integrity and catchment integrity throughout each entire watershed (Figure 4). The combined distribution of IWI and ICI values throughout each entire watershed (i.e., not limited to only sampled sites within each watershed) was normally distributed and ranged from a low of 0.19 to a high of 1.0 (SD = 0.15) for IWI, and from a low of 0.13 to a high of 1.0 (SD = 0.16) for ICI (Table 6). The distribution of the IWI and ICI values at the sampled sites within each watershed were generally normally distributed, except for the Calapooia watershed which exhibited a more bimodal distribution (Figure 5).

Each of the case study watersheds exhibited a more limited range of IWI and ICI values when considered for the specific sampling sites where response variables were obtained. The lowest IWI/ICI values for sampled sites of 0.40 and 0.39, respectively, were in the Choptank study area watershed and the highest IWI/ICI values of 0.98 and 0.98, respectively, were in the Calapooia watershed (Table 6). The median IWI value at sampled sites for the four case study watersheds combined was 0.59 (SD = 0.16), with median IWI values ranging from a low of 0.49 (SD = 0.12) for the East Fork Little Miami River to a high of 0.88 (SD = 0.20) for the Calapooia River watershed. The largest variation in IWI values across the four watersheds occurred in the Calapooia (SD = 0.20), while the smallest variation in IWI values was found in the Choptank study area watershed (SD = 0.07). The summary statistics for the ICI values were similar to the IWI for the four watersheds combined, with a slightly higher median ICI value of 0.61 (SD = 0.17), compared to the median IWI value of 0.59 (SD = 0.16). Comparing across the four watersheds the median ICI values at sampled sites ranged from a low of 0.58 (SD = 0.13) for the Choptank study area watershed to a high of 0.75 (SD = 0.12) for the Narragansett Bay watershed (Table 6).

### 3.2. Correlations with IWI/ICI Values Across Case Study Watersheds

Of the 29 response variables from the four case study watersheds, 15% of the response variables demonstrated ‘very strong’ (0.80–1.0) correlations, 26% demonstrated ‘strong’ (0.60–0.79) correlations, 32% exhibited ‘moderate’ (0.40–0.59) correlations, 15% revealed ‘weak’ (0.20–0.39) correlations and 12% of the response variables demonstrated ‘very weak’ (0.0–0.19) correlations with IWI/ICI values and their associated six functional component indices (Tables 7–10). Results for specific response variables are discussed by watershed below.

#### 3.2.1. Calapooia River Watershed

**Figure 6:**
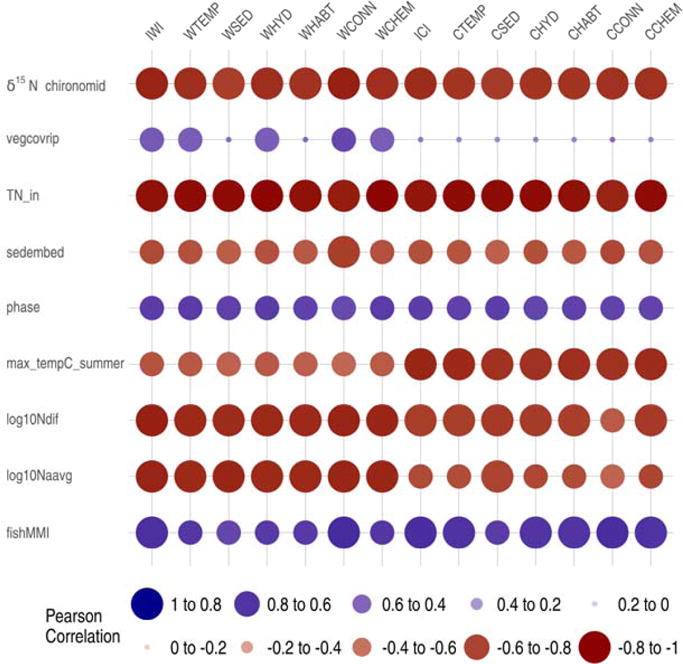
Calapooia River Watershed response variable correlations with Indices of Watershed (IWI) and Catchment Integrity (ICI) and associated six functional component indices. Functional components at watershed (w) and catchment (c) scales: HYD = hydrologic regulation; CHEM = regulation of water chemistry; SED = sediment regulation; CONN = hydrologic connectivity; TEMP = temperature regulation; and HABT = habitat provision. See Table 7 for key response metric descriptions. Dot size proportional to relative magnitude of correlation and color indicates direction of correlation.

All the various response variables measured in the CRW were highly significantly correlated to the IWI/ICI values along with their respective functional components (Figure 6, Table 7). The response metric, total_in (annual total nitrogen (TN) input), an index that quantifies TN input calculated from all anthropogenic and natural sources at a watershed scale (n = 13), had the strongest negative linear correlation with IWI and ICI values, with a Pearson’s correlation coefficient (*r*), of −0.97 and −0.96, respectively, indicating that as TN input increases IWI and ICI decreases. All six of the associated functional component indices for IWI and ICI were also significantly correlated with TN input with |*r*| values ranging from 0.92 for CCONN (hydrological connectivity at catchment scale) to 0.99 for both WCHEM (chemical regulation at watershed scale) and WHYD (hydrologic regulation at watershed scale) (Figure 6, Table 7). Another response variable exhibiting highly significant negative correlations with IWI (*r* = −0.93) and ICI (*r* = −0.82) values is the metric log10Ndif, which measures the log of the fluctuation in annual stream nitrate concentration from 54 streams within the Calapooia watershed (Figure 6). The monthly average nitrate concentrations in these streams (log10Navg) also demonstrated significantly negative correlations with IWI (*r* = −0.92) and ICI (*r* = −0.77) (Table 7). Measured nitrogen-stable isotope ratios (δ^15^N) in chironomids collected from 22 streams within the Calapooia watershed also demonstrated a highly significant negative correlation with IWI (*r* = −0.92) and ICI (*r* = −0.89) values (Figure 6, Table 7). The fish MMIs developed in the Calapooia watershed demonstrate a highly significant positive correlation with IWI and ICI values (*r* = 0.82 and *r* = 0.83, respectively). Four additional stream response metrics including percent riparian vegetation cover (vegcovrip), sediment embeddedness (sedembed), maximum summer stream temperature (max_tempC_summer) and index of thermal regime timing (phase), all exhibited strong correlations with IWI values with |*r*| values ranging from 0.65 (vegcovrip) to 0.77 (phase and sedembed) and ICI values ranging from 0.52 (vegcovrip) to 0.91 (max tempC_summer). All the six functional components associated with the IWI and ICI values demonstrated similarly strong correlations for each corresponding response variable metric (Table 7). Several other response variable indices quantifying total nitrogen input, export, and retention, which were calculated as part of the Calapooia Watershed Nitrogen budget project [37], also exhibit highly significant negative correlations with IWI and ICI values, and are shown in the Supplementary Materials (Table S1).

#### 3.2.2. Choptank Watershed

**Figure 7:**
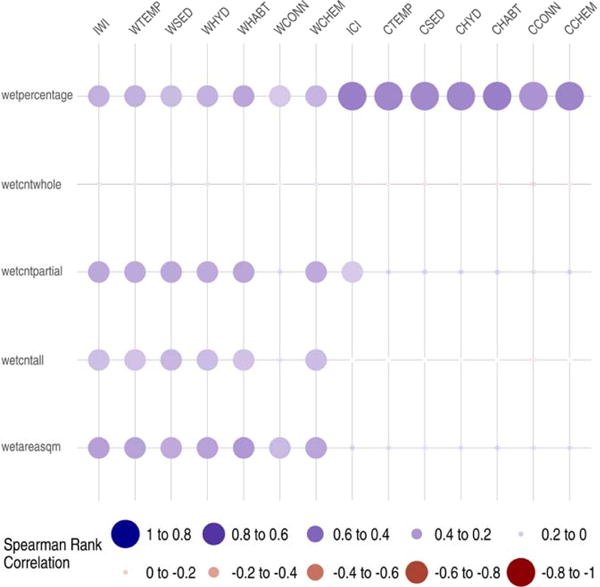
Choptank Watershed study area response variable correlations with Indices of Watershed (IWI) and Catchment Integrity (ICI) and associated six functional component indices. Functional components at watershed (w) and catchment (c) scales: HYD = hydrologic regulation; CHEM = regulation of water chemistry; SED = sediment regulation; CONN = hydrologic connectivity; TEMP = temperature regulation; and HABT = habitat provision. See Table 8 for key response metric descriptions. Dot size proportional to relative magnitude of correlation and color indicates direction of correlation.

In the Choptank watershed study area the response variables developed using both the NHDPlusV2 and the semi-automated LiDAR derived stream networks were not normally distributed, so Spearman’s rank correlation coefficients were used to measure the strength of the association between the response variables and IWI/ICI values. The results were similar for the two different methods characterizing stream networks producing slightly higher correlations with the IWI/ICI values for the high resolution semi-automated LiDAR stream representation. We describe here the results using the NHDPlusV2 method and provide the results with semi-automated LiDAR in Supplementary Materials (Table S2). The five response variable metrics developed to represent hydrologic connectivity by quantifying the intersecting wetland-stream connectivity varied in their correlation with IWI and ICI values. For some correlations the response variable metrics were more strongly associated with the ICI and catchment-scale functional component indices than with the IWI and watershed-scale functional component indices. The metric percentage of areal coverage for wetland polygons intersecting stream channels in a catchment (wetpercentage) demonstrated the strongest positive correlation with ICI (r_s_ = 0.49) while the area of wetland intersecting stream channels in the catchment metric (wetareasqm) demonstrated the strongest positive correlation with IWI (*r*_*s*_ = 0.36) (Figure 7, Table 8). The three other response metrics that quantified the number of wetland polygons at the catchment scale demonstrated weak correlations. The six functional components associated with the IWI and ICI values demonstrated similar strengths and direction of correlation with each of the response variable metrics (Figure 7, Table 8).

#### 3.2.3. East Fork Little Miami River Watershed

**Figure 8:**
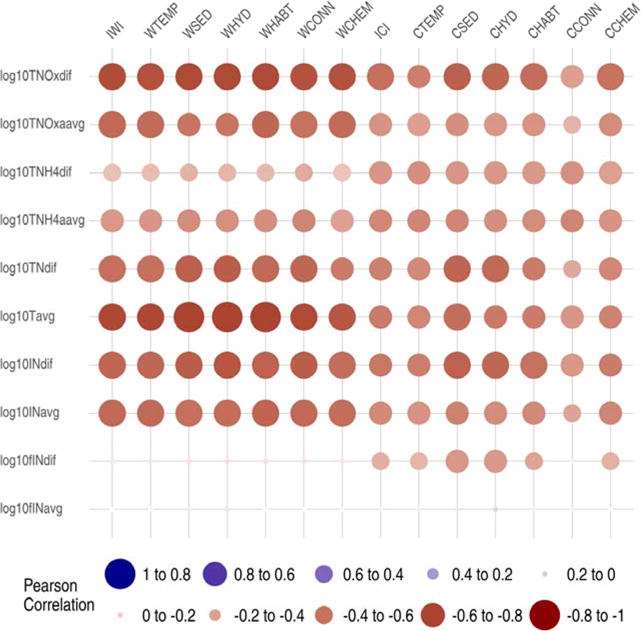
East Fork Little Miami River Watershed response variable correlations with Indices of Watershed (IWI) and Catchment Integrity (ICI) and associated six functional component indices. Functional components at watershed (w) and catchment (c) scales: HYD = hydrologic regulation; CHEM = regulation of water chemistry; SED = sediment regulation; CONN = hydrologic connectivity; TEMP = temperature regulation; and HABT = habitat provision. See Table 9 for key response metric descriptions. Dot size proportional to relative magnitude of correlation and color indicates direction of correlation.

Eight of the ten nitrogen metrics measured in the EFLMR demonstrated significantly negative correlations with IWI, while nine of the ten were significant for ICI. The strongest negative correlations were associated with annual average of total nitrogen (log10Tavg) and IWI (r = −0.78), and the annual range in total nitrate/nitrite (log10TNoxdif) and ICI (r = −0.61). All the IWI and ICI associated six functional component indices demonstrated similar strengths and correlations with each of the nitrogen metrics (Figure 8, Table 9). The only nitrogen metric that did not demonstrate any significant correlation with the IWI and ICI values was the fraction of Total-N as Inorganic-N (log10fINavg) and the annual range in the fraction of Total-N as Inorganic-N (log10fINdif) was not significantly correlated with the IWI.

#### 3.2.4. Narragansett Bay Watershed

**Figure 9:**
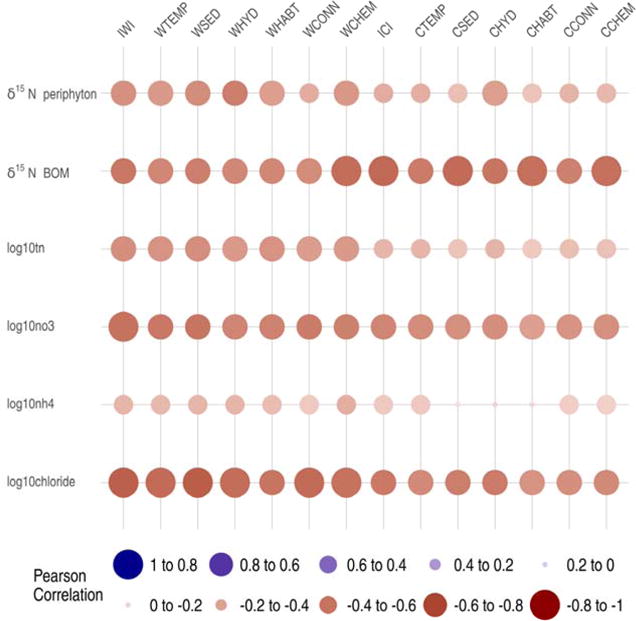
Narragansett Bay Watershed response variable correlations with Indices of Watershed (IWI) and Catchment Integrity (ICI) and associated six functional component indices. Functional components at watershed (w) and catchment (c) scales: HYD = hydrologic regulation; CHEM = regulation of water chemistry; SED = sediment regulation; CONN = hydrologic connectivity; TEMP = temperature regulation; and HABT = habitat provision. See Table 10 for key response metric descriptions. Dot size proportional to relative magnitude of correlation and color indicates direction of correlation.

The nitrogen (TN, NO_3_-N and NH_4_^+^) and watershed development metrics (Cl^−^) measured in streams within NBW all demonstrated significant negative correlations with IWI, ICI and their six associated functional component indices. Log10 chloride concentrations had the strongest negative correlations with IWI and ICI with *r* = −0.68 and *r* = −0.57, respectively. Of the nitrogen metrics, log10 NO_3_-N concentrations exhibited the strongest negative correlation with IWI (*r* = 0.60) and ICI (*r* = −0.52) values. The measured nitrogen-stable isotope ratios (δ^15^N) of periphyton (*pN15*) also demonstrated a significant negative correlation with IWI and ICI values with *r* = 0.47 and *r* = 0.35, respectively (Figure 9, Table 10). All the six functional components associated with IWI and ICI showed similar relationships as with the IWI and ICI correlations for each response variable (Figure 9, Table 10).

The measured δ^15^N of BOM (*d15NBOM*) collected from lakes showed a significantly negative correlation with IWI and ICI values with *r* = −0.58 and *r* = −0.64, respectively. All the six functional components associated with IWI and ICI showed similar relationships as with the IWI and ICI correlations for the δ^15^N BOM (Figure 9, Table 10).

### 3.3. Correlations with Landscape Explanatory Variables Across Case Study Watersheds

The correlation strength of individual landscape explanatory variables (%Urb, %Agr and %For) associated with response variables at the watershed and catchment scales varied widely across locations (Table 11). However, the IWI performed well at all four locations: the average of the percent of the maximum absolute correlation at the watershed scale for the IWI was 99.7, 80.3, 92.6, and 100.0% at CRW, EFLMR, NBW, and CHOP, respectively, and 89.4% overall. Similar values for the ICI were 99.3, 83.4, 83.5, and 50.4%, respectively, and 79.7% overall—indicating weaker performance at NBW and especially CHOP. The IWI and ICI had the highest rank in absolute correlation results for 47.8% and 26.1% of the 23 response variable comparisons, respectively, and they were ranked in the top two for 78.3% and 73.9% of the comparisons, respectively (note: these numbers included ties, e.g., ranks of 1.5 or 2.5). These percentages of high rankings equal or exceed those of each of the other three landscape variables, except in one case: %For had the highest rank in 43.5% of the comparisons for the ICI. In pairwise comparisons of the absolute correlations between the integrity index and each landscape variable, the IWI absolute correlations exceeded the other variables in 66.7% of the 69 comparisons, while the ICI exceeded the absolute correlations of these variables in 59.4% of the cases. At the watershed scale, if the variable with the maximum absolute correlation was not the IWI, then usually that dominant landscape variable did not vary by location. For example, in cases where IWI did not have the maximum absolute correlation at EFLMR and NBW, %Agr and %For, respectively, had the highest absolute correlations in all but one case (Table 11). In contrast, dominant landscape variables were more varied at the catchment scale when the ICI did not have the maximum absolute correlation; e.g., %Urb, %For, and %Agr all dominate at EFLMR with respect to different response variables.

## 4. Discussion

In this study, we examine how a range of response variables measured in four watersheds in four different ecoregions across the CONUS that have different geophysical and ecological conditions compare with national indices of integrity. These response variables varied across case study watersheds thematically (i.e., chemical, biological, habitat) and were measured over varying temporal scales (i.e., single measure, monthly, multi-year), spatial scales, and ecosystems (streams, lakes, and wetlands). Using these independently collected response variables across characteristically different watersheds allows us to evaluate the robustness of this national index of watershed integrity.

Comparing response variable correlations with IWI/ICI values within each case study watershed, the CRW had the highest percentage of *very strong* (0.80–1.0) correlations with 57% of the response variables significantly correlated with IWI/ICI values. The CRW nitrogen-related response metrics were all significantly and negatively correlated with the IWI/ICI values, with |r| for IWI ranging from 0.92 to 0.97, and 0.82 to 0.96 for ICI. Comparing nitrogen-related response metrics across watersheds (CRW, EFLMR, and NBW) with varying sources of nutrients (e.g., CRW and EFLMR: agricultural fertilizer; NBW: urban wastewater, surface runoff, leaking onsite wastewater treatment systems), and varying temporal scales (single summer sampling event, monthly, seasonal, annual averages, etc.), it is noteworthy that the majority (74%) of nitrogen-related metrics exhibited significantly moderate to very strong (|r| = 0.40–1.0), negative linear correlations with IWI and ICI values. Comparing site level response metrics across watersheds, for example, NO_3_ concentration, which was measured at three of the four watersheds, reveals a pattern of lower correlation strength with the IWI as the size of the watershed increases (e.g., correlation strength with IWI with NO_3_: NBW < EFLMR < CRW). The IWI exhibiting higher correlations with stressors in systems where there are fewer risk factors could be related to the complex and highly variable nature of antagonistic interactions where multiple stressors (risk factors) exist. As systems get bigger (% open water, river km, watershed size) their degree of disturbance increases (% urban, % imperviousness, population density), and as disturbance increases, the correlations with IWI decrease.

Another nitrogen-related response metric that demonstrated strong negative correlations with IWI and ICI values that can be compared across watersheds is the measured nitrogen-stable isotope ratios of chironomids (in CRW streams), periphyton (in NBW streams), and benthic organic matter (in NBW lakes). Again, the nitrogen sources varied (agricultural to urban), as well as the aquatic ecosystems (streams, lakes), and temporal scales (single summer-time sample event, repeated measures, seasonal, annual averages), yet 83% of the nitrogen-stable isotope ratio response measures revealed moderate to very strong negative correlations (*r* = 0.40–1.0) with IWI and ICI values. Stable isotope ratios of periphyton and stream macroinvertebrates (e.g., chironomids) have been shown to be effective indicators of watershed development effects on stream ecosystems [67,68]. This can also make them useful for quantifying the effectiveness of nitrogen, stream, and watershed management efforts [62]. The strong relationship between these nitrogen-stable isotope ratios and IWI and ICI values across watersheds demonstrates the integrative nature of the IWI.

In the Choptank watershed study area, response metrics were developed specifically to represent and quantify wetland connectivity to streams by measuring the number and percentage of streams intersecting non-riverine wetlands using two different methods for characterizing stream networks: finer resolution semi-automated LiDAR, and NHDPlusV2. Both methods resulted in similar relationships with IWI and ICI, and in both cases the ICI was more strongly correlated with the percentage of areal coverage for wetland polygons intersecting stream channels in a catchment (wetpercentage). Relative to issues of data source resolution, it is reaffirming that the correlation relationships with IWI and ICI values were very similar regardless of the resolution of stream characterization method. The wetpercentage response variable developed for the Choptank watershed study area is the variable that most pragmatically represents aquatic connectivity as it quantifies the connection between streams and wetlands within each catchment. Considering that the Choptank watershed study area is essentially at sea level with very little elevation, it makes intuitive sense that the catchment values (ICI and associated six functional component indices) would be more closely correlated as the catchment would be more influential absent any effects from upstream watersheds.

In a previous study, Thornbrugh et al. [24] used site-scale indicators from the US EPA’s 2008–2009 NRSA survey, which sampled 1924 perennial streams across CONUS using a spatially balanced sampling design to develop national and regional estimates of stream condition. The site-scale indicators included water quality metrics (based on total nitrogen, total phosphorus, and turbidity), stream physical habitat and biological indicators. Using simple regression analyses (using site indicators as response variables and IWI/ICI as independent variables) they found that IWI accounted for 27% of the national variation in the water quality indicator for the CONUS, and only 2–12% of the variation in the biological and stream physical habitat indicators for the CONUS [24] (see Tables S7 and S5 in [24]). In this study, using response data from more intensively sampled watersheds, we find a higher percentage of the variation explained by IWI and ICI. For example, using the R^2^ values for the 18 nitrogen response metrics (including stable isotope ratios, δ^15^N), IWI accounts for 39% and ICI accounts for 32% of the variation in nitrogen response across the Calapooia River, East Fork Little Miami River and Narragansett Bay watersheds. In the Calapooia River watershed, IWI accounts for 88% and ICI accounts for 80% of the variation in the nitrogen response metrics.

Thornbrugh et al. [24] also analyzed the correlation between the water quality indicator (derived from total nitrogen, total phosphorus, and turbidity concentrations measured at NRSA sites from 2008–2009) and landscape indicators (% urban and % agriculture) at catchment and watershed scales for each NRSA site. At the CONUS scale, IWI exhibited the highest linear relationship (adjusted R^2^ = 0.27) with the water quality indicator, followed by the % agriculture at the watershed scale (adjusted R^2^ = 0.25), while the % urban at the watershed scale exhibited a much weaker linear relationship (adjusted R^2^ = 0.01). These analyses demonstrated that there was an added benefit or value in applying the watershed integrity index to explain the variance in water quality, versus using individual landscape indicators to characterize aquatic condition (see Table S7 in [24]). In this study, the integrity indices clearly outperformed three individual landscape variables (%Urb, %For, %Agr), especially at the watershed-scale. IWI and ICI absolute correlations were 80–100% and 50–99% of maximum correlations, respectively. The percent of cases where the IWI or ICI had either the highest rank or was ranked in the top two exceeded the percentages for the other three landscape variables, except that %For was highest ranked for more of the ICI comparisons. The absolute correlations of the IWI and ICI exceeded those of the other three landscape variables in 67 and 59% of pairwise comparisons, respectively. Finally, while each of the three landscape variables outperformed the IWI or ICI for specific response variables, none of these performed consistently well across all four locations. This is a critical shortcoming for any analysis at national or large regional scales. For example, while %Agr would be a better overall indicator than the IWI for EFLMR watersheds, it performed poorly at NBW. This is because the individual landscape variables cannot perform well in areas where they do not have a wide variance. In contrast, the IWI and ICI performed well across all four locations. These two indices are more robust because they incorporate multiple stressors, and so have a higher likelihood of being relevant to a diversity of landscapes.

Recently Aho et al. [69] conducted a study adapting and applying this index of watershed integrity approach to the Western Balkans’ transboundary river and lake basins region. Aho et al. demonstrated that this concept of watershed and catchment integrity can be successfully transferred to other countries, regions, and watersheds by using comparable data coverages (to quantify human-related stressors as in Table 3 of this study) that were available for the entire Balkans study area to develop the IWI and ICI values for the Balkans. This adaptation of the index of watershed/catchment integrity to the Balkans also demonstrates how local, national, and international entities and watershed managers can use the mapped stressor, functional component, and ICI and IWI information at multiple scales of governance. One of the suggested applications of the IWI and ICI described by Aho et al. [69] is to focus management efforts in areas where there are catchments with low integrity ICI values located within watersheds with high integrity IWI values. This approach was based on an application developed in the CONUS by Hill et al. [70]. By deconstructing the six functional components within a catchment with low ICI values, watershed managers or local entities can focus on the functional elements and associated stressors contributing to the degraded aquatic functions. Using the maps of IWI and ICI to prioritize restoration and conservation efforts within catchments that have high watershed integrity values increases the probability for achieving positive impact on the functional components of integrity. Deconstruction and evaluation of the indices and their associated risk factors can help to target management efforts at multiple scales of governance from local town or municipal scales to watershed scales that cross trans-political boundaries.

Within the US there are opportunities to apply this watershed integrity approach in coordination with the US EPA’ s Healthy Watershed Program which uses integrated assessments ranging from screening-level assessments using GIS data layers to statistical and geospatial modeling of ecological attributes (https://www.epa.gov/hwp/download-2017-preliminary-healthy-watersheds-assessments). The IWI and ICI can also be applied in conjunction with the US EPA’s Recovery Potential Screening (RPS) (https://www.epa.gov/rps) which provides technical methods and tools for comparing large numbers (e.g., hundreds to thousands) of hydrologic unit codes (HUC) based on the USGS HUC framework, a dataset based on drainage subdivisions of land surface areas at several hierarchical levels [71]. The RPS tools measure several ecological, stressor and social indicators for each HUC that are associated with the likelihood that a HUC is in reasonably good condition and a protection or restoration effort may succeed. Combining the RPS with the IWI approach would have the advantage of incorporating true watershed information from the IWI into the restoration analysis, since HUCs do not integrate the upstream area. Applying the IWI and ICI approach along with the EPA’s RPS provides watershed managers with a systematic approach for targeting restoration within watersheds while considering social factors such as community involvement, incentives, economics, governance, regulation, and planning status, which may strongly influence the level of effort and complexity of achieving improvements.

The results from this evaluation provide strong support for the utility of applying indices of watershed and catchment integrity derived from nationally available data to identify areas of high integrity to target protection and alternatively to identify impacted areas for focusing regional and watershed level restoration efforts. Considering the wide range of site-level response variable metrics and indices and the very different characteristics of the case study watersheds, finding 41% of the response variables across watersheds strongly correlated with IWI and ICI values, with |*r*| ranging from 0.60–1.0, and an additional 32% with moderate |*r*| values of 0.40–0.59, provides significant weight-of-evidence supporting the validity of this national-scale extent mapping and assessment of watershed integrity.

A national map of watershed integrity could be of value to states, tribal, regional, and local watershed organizations that are initiating healthy watershed programs, implementing systems-based healthy watershed protection by identifying and prioritizing conservation and protection of watersheds with high integrity, as well as identifying functional components within watersheds that have good potential for restoration and rehabilitation efforts. National maps of watershed and catchment integrity provide a broad landscape perspective and context by revealing the condition of terrestrial and aquatic landscapes adjacent to and upstream of focal areas, providing stakeholders and decision-makers with valuable information that can be used for multi-scale approaches of protection and restoration [70]. This initial evaluation of the national indices of integrity with independently collected and developed site-level response metrics strengthens the rationale for using national metrics of watershed integrity combined with local data to address water resource management decisions across multiple scales of governance.

## 5. Conclusions

One of the main objectives of the US Clean Water Act (CWA), is “to restore and maintain the chemical, physical, and biological integrity of the nation’s waters”. The intent of the term “integrity” in the CWA was to recognize the importance of preserving natural ecosystems that support key watershed processes, water quality, and the condition of aquatic ecosystems [1]. Much of the nation’s federal and state-level water quality programs over the past four decades have focused on identifying and restoring impaired waters and reducing point and non-point sources of pollution entering waterways. Many of these federal, tribal, state, and non-governmental entities recognize the dual ecological and economic benefits of protecting healthy functioning watersheds while avoiding expensive and not entirely effective restoration of waterbodies (US EPA Healthy Watersheds Program: https://www.epa.gov/hwp).

This study demonstrates that the two integrity indices are related to site-scale response variables for streams, lakes, and wetlands and across four study areas that vary in geophysical and ecological conditions. In contrast to three other landscape variables, the integrity indices are robust since they incorporate multiple stressors and so perform well across all four locations. An empirically based, national-scale mapping and assessment of watershed integrity provides EPA and state programs with additional information for fulfilling objectives of the CWA and supporting tenets of the Healthy Watersheds Program by providing a consistent and systematic geospatial framework for evaluating watershed integrity based on risks to key watershed functions. A nationally consistent map of watershed integrity facilitates comparisons among regions and can assist states and other agencies in identifying and prioritizing protection for healthy watersheds, as well as targeting critical functional elements of watersheds for restoration efforts.

## Supplementary Material

Supplement1

## Figures and Tables

**Table 1:** Key functions that occur in unaltered watersheds and the major stressors affecting these functions.

Key Function	Description	Major Stressors	
		Within Channel	Outside Channel
HYD	Maintenance of the natural timing, pattern, supply, and storage of water that flows through the watershed	Presence and volumes of reservoirs (NABD) Stream channelization and levee construction (NA)	Percent of the watershed comprising agricultural land use (NLCD) Total length and density of canals/ditches (NHD) Percent imperviousness of human-related landscapes (NLCD) Alteration to and spatial arrangement of riparian vegetation (LANDFIRE) Boundaries, depths, and flows of aquifers (NA) Groundwater use (NA) *
CHEM	Maintenance of the natural timing, supply, and storage of the major chemical constituents of freshwaters: nutrients (nitrogen and phosphorus), salinity or conductivity, total dissolved solids, hydrogen ions (pH), and naturally occurring minor constituents (e.g., heavy metals). Human-related alterations can include deviations from naturally occurring concentrations of these constituents or the inclusion of non-naturally occurring constituents, such as pesticides and industrial chemicals.	Presence and volumes of reservoirs (NABD) Stream channelization and levee construction (NA)	Atmospheric deposition of anthropogenic sources of nitrogen and acid rain (NADP) Percent of watershed composed of urban and agricultural land uses (NLCD) Fertilizer application rates (FERT) Presence and density of wastewater treatment facilities (NPDES), industrial facilities (TRI), superfund sites (SUPERFUND), and mines (MINES) Cattle density (NA) * Alteration to and spatial arrangement of riparian vegetation (LANDFIRE) Chemical constituents of groundwater (NA)
SED	Maintenance of the volume and size composition of inorganic particles that are stored or transported through the stream or within lakes, wetlands, or estuaries.	Presence and volumes of reservoirs (NABD) Stream channelization and levee construction (NA)	Alteration to and spatial arrangement of riparian vegetation (LANDFIRE) Presence and density of mines (MINES), forest cover loss (GFC), and roads (TIGER) Agriculture (NLCD) weighted by soil erodibility (CONUS-SOIL)
CONN	Presence of hydrologic pathways for the transfer of matter, energy, genes, and organisms within watersheds. Systems can vary naturally in their hydrologic isolation (e.g., desert springs) or connectedness (e.g., the Everglades).	Presence and volumes of reservoirs (NABD) Stream channelization and levee construction (NA) Road/stream intersections (TIGER/NHD) weighted by stream reach slope (NHD)	Alteration to and spatial arrangement of riparian vegetation (LANDFIRE) Density of ditches/canals (NHD) Groundwater use (NA) * Presence and density of wastewater discharge sites (NPDES) Percent of riparian zone composed of urban and agricultural land uses (NLCD)
TEMP	Maintenance of the full range of natural landscape features (both aquatic and terrestrial) required to maintain temperatures that support the aquatic chemistry and biota.	Presence and volumes of reservoirs (NABD)	Alteration to and spatial arrangement of riparian vegetation (LANDFIRE) Percent of watershed composed of agricultural land uses (NLCD) Percent of watershed composed of urban land uses in the riparian zone (NLCD) Groundwater use (NA) * Presence and density of wastewater discharge sites (NPDES)
HABT	Presence and maintenance of the full range of natural landscape features (both aquatic and terrestrial) that represent the complete set of conditions that are needed to maintain the natural diversity and abundances of aquatic biota.	Presence and volumes of reservoirs (NABD)	Alteration to and spatial arrangement of riparian vegetation (LANDFIRE) Density of housing unit developments within riparian zones (TIGER) Percent of watershed composed of agricultural land uses (NLCD) Density of road/stream intersections (TIGER/NHD) Density of roads within riparian zones (TIGER)

Data sources that can be used to evaluate the stressors are included parenthetically (see key at bottom of table). Within each function highly correlated stressors (correlation coefficients *r* > 0.7) were eliminated. Table adapted from Flotemersch et al. [2] and Thornbrugh et al. [24].

CONUS-SOIL—Penn State University soil characteristics dataset, based on STATSGO (http://www.soilinfo.psu.edu/index.cgi?soil_data&conus); FERT—County-level estimates of N and P from commercial fertilizer (http://pubs.usgs.gov/sir/2012/5207); GFC—University of Maryland Global Forest Change 2000–2013 Dataset (http://earthenginepartners.appspot.com/science-2013-global-forest/download_v1.1.html); LANDFIRE—USFS and USDOI LANDFIRE Program (http://www.landfire.gov); MINES—USGS Mines Dataset (https://www.sciencebase.gov/catalog/ folder/4f4e4767e4b07f02db47e0ad), USGS National Coal Resources Data System (NCRDS), and US Stratigraphy (USTRAT) data of coal mine sites (http://ncrdspublic.er.usgs.gov/ncrds_data); NA—Not available; NADP—National Atmospheric Deposition Program National Trends Network (http://nadp.sws.uiuc.edu/data/ntn); NHD—National Hydrography Dataset (http://www.horizon-systems.com/NHDPlus/NHDPlusV2_home.php); NABD—2012 National Anthropogenic Barrier Dataset (https://www.sciencebase.gov/catalog/item/56a7f9dce4b0b28f1184dabd); NLCD—National Land Cover Dataset (http://www.mrlc.gov/nlcd06_data.php); NPDES—USEPA National Pollutant Discharge Elimination System (http://www.epa.gov/enviro/geo_data.html); SUPERFUND—USEPA Superfund Sites (http://www.epa.gov/enviro/geo_data.html); TIGER—US Census Bureau TIGER/Line Program (http://www2.census.gov/geo/pdfs/maps-data/data/tiger/tgrshp2013/TGRSHP2013_TechDoc.pdf); TRI—National Toxic Release Inventory (http://www.epa.gov/enviro/geo_data.htm);

* County data were available for groundwater use and cattle density but were not utilized because of quality control and data resolution issues.

**Table 2:** Human-related landscape stressors associated with six watershed functional components used to develop the Index of Watershed Integrity (IWI).

Variable	HYD	CHEM	SED	CONN	TEMP	HABT
PctUrb2006Ws		X				
PctAg2006Ws	X	X			X	X
PctImp2006Ws	X					
RdDensWs			X			
RdCrsWs						X
NABD_DensWs	X	X	X	X	X	X
NABD_NrmStorWs	X	X	X	X	X	X
AgKffactWs			X			
MineDensWs		X	X			
CoalMineDensWs		X	X			
CanalDensWs	X			X		
RdCrsSlpWtdWs				X		
InorgNWetDepWs		X				
FertWs		X				
NPDESDensWs		X		X	X	
TRIDensWs		X				
SuperfundDensWs		X				
PctUrb2006WsRp100				X	X	
PctAg2006WsRp100				X		
PctNonAgIntrodManag	X	X	X	X	X	X
PctFrstLoss2006Ws			X			
RdDensWsRp100						X
HUDens2010WsRp100						X

HYD: hydrologic regulation; CHEM: regulation of water chemistry; SED: sediment regulation; CONN: hydrologic connectivity; TEMP: temperature regulation; HABT: habitat provision. See Table 3 for full variable description. Figure adapted from Flotemersch et al. [2] and Thornbrugh et al. [24].

**Table 3:** Description of 23 human-related landscape stressors used to develop the Index of Watershed Integrity (IWI).

Variable Name	Description
PctUrb2006Ws	% of watershed area classified as developed, high, medium, and low-intensity land use (NLCD 2006 class 22, 23, 24)
PctAg2006Ws	% of watershed area classified as crop and hay land use (NLCD 2006 class 81 and 82)
PctImp2006Ws	% imperviousness of anthropogenic surfaces within watershed
RdDensWs	Density of roads (2010 Census Tiger Lines) within watershed (km/km^2^)
RdCrsWs	Density of roads-stream intersections (2010 Census Tiger Lines-NHD stream lines) within watershed (crossings/km^2^)
NABD_DensWs	Density of georeferenced dams within watershed (dams/km^2^)
NABD_NrmStorWs	Volume all reservoirs (NORM_STORA in NID) per unit area of watershed (cubic meters/km^2^)
AgKffactWs	The Kffact is used in the Universal Soil Loss Equation (USLE) and represents a relative index of susceptibility of bare, cultivated soil to particle detachment and transport by rainfall within watershed
MineDensWs	Density of mines sites within watershed (mines/km^2^)
CoalMineDensWs	Density of coal mines within the watershed (mines/km^2^)
CanalDensWs	Density of NHDPlus line features classified as canal, ditch, or pipeline within the upstream watershed (km/km^2^)
RdCrsSlpWtdWs	Mean stream slope (NHD stream slope) of roads-stream intersections (2010 Census Tiger Lines-NHD stream lines) within watershed (crossings/km^2^)
InorgNWetDepWs	Annual gradient map of precipitation-weighted mean deposition for inorganic nitrogen wet deposition from nitrate and ammonium for 2008 in kg of NH_4_^+^ ha/year, within watershed
FertWs	Mean rate of synthetic nitrogen fertilizer application to agricultural land in kg N/ha/year, within watershed
NPDESDensWs	Density of permitted NPDES (National Pollutant Discharge Elimination System) sites within watershed (sites/km^2^)
TRIDensWs	Density of TRI (Toxic Release Inventory) sites within watershed (sites/km^2^)
SuperfundDensWs	Density of Superfund sites within watershed and within 100-m buffer of NHD stream lines (sites/km^2^)
PctUrb2006WsRp100	% of watershed area classified as developed, high, medium, and low -intensity land use (NLCD 2006 class 22, 23, 24) within a 100-m buffer of NHD streams
PctAg2006WsRp100	% of watershed area classified as crop and hay land use (NLCD 2006 class 81 and 82) within a 100-m buffer of NHD streams
PctFrstLoss2006Ws	% Forest cover loss (Tree canopy cover change) for 2006 within watershed
PctNonAgIntrodManag VegWsRp100	% Non-agriculture non-native introduced or managed vegetation landcover type reclassed from LANDFIRE Existing Vegetation Type (EVT), within watershed and within 100-m buffer of NHD stream lines
RdDensWsRp100	Density of roads (2010 Census Tiger Lines) within watershed and within 100-m buffer of NHD stream lines (km/km^2^)
HUDen2010WsRp100	Mean housing unit density (housing units/km^2^) within watershed and within a 100-m buffer of NHD stream lines

The same 23 stressors are used in developing the Index of Catchment Integrity (ICI), except the values are calculated at the catchment scale.

**Table 4:** Case study watershed landscape and demographic characteristics.

Watershed	Calapooia River	Choptank Study Area	East Fork Little Miami River	Narragansett Bay
Size (km^2^)	945	1070	1293	4421
Elevation range (m)	57–1562	0–36	149–365	0–423
Land Use Classification a				
% Agriculture	52.6	59.5	55	6.3
% Forest	28.7	11.7	32	38.9
% Brushland	11.8	1.2	0.27	0.98
% Urban/Developed	5.11	6.4	11.4	34.7
% Wetland	1.6	20.4	0.16	14.9
% Open Water	0.07	0.8	1.1	3.4
% Impervious Surface	1.82	0.85	2.51	14.96
Population b (2010)	28,959	37,164	129,670	1,962,003
Population Density (Persons/km^2^)	30	35	100	442
Annual Average Precipitation c (cm)	145	111	108	125
River Kilometer (km)	619	812	1251	2489

a Land Use Classification based on 2011 NLCD;

b [25];

c PRISM spatially gridded average annual precipitation at 800 m grid cell resolution. Data derived from monthly 30-year “normal” dataset covering the conterminous U.S., averaged over the climatological period 1981–2010. http://www.prism.oregonstate.edu/normals/.

**Table 5:** Description of response variables for each case study watershed.

Type	Response Variable	Response Variable Description	Sample Sites (n)
		CRW	

S	δ^15^N chironomid	Chironomid nitrogen isotopic composition (δ^15^N‰) collected from 2013–2015	22
S	log10Naavg	Log10 of the average NO_3_ concentrations of each month over the entire sampling period were calculated at each site.	53
S	log10Ndif	Ndif, calculated as the difference between the highest and lowest monthly values of log10 NO_3_ concentrations at each site.	53
S	TN_in (kg N/ha/year)	Annual total nitrogen (TN) input from all anthropogenic and natural sources. Indices were calculated in the Calapooia River Watershed N budget project [37] see Supplementary Materials.	13
S	TN_out (kg N/ha/year)	Annual total nitrogen (TN) export using LOADEST model (U.S. Geological Survey, Reston, VA, USA). Indices were calculated in the Calapooia River Watershed N budget project [37] see Supplementary Materials.	13
S	ag_frt (kg N/ha/year)	Annual TN input from fertilization. Indices were calculated in the Calapooia River Watershed N budget project [37] see Supplementary Materials.	13
S	winter_frt (kg N/ha)	Fertilizer N input in winter. Indices were calculated in the Calapooia River Watershed N budget project [37] see Supplementary Materials.	13
S	harvest (kg N/ha/year)	Annual N removal via crop harvest. Indices were calculated in the Calapooia River Watershed N budget project [37] see Supplementary Materials.	13
S	resN (kg N/ha/year)	Retention N—difference between annual TN input and annual TN export. Indices were calculated in the Calapooia River Watershed N budget project [37] see Supplementary Materials.	13
S	fishMMI	Fish MMIs were calculated following methods set by Whittier et al. [45]. Seven metrics for the Western Mountains Ecoregion were selected and consisted of an assemblage tolerance index estimating overall resilience to disturbance [45]. Scores were generated for each unique sampling event and a mean inter-annual score was attributed to sites receiving multiple visits over the course of the study. Final scores were rescaled to values between 0 and 100 for comparison with the IWI, with increasing scores indicating higher overall ecological condition. Sampling occurred throughout the year but for consistency only NRSA index period data were used for this analysis (summer low-flow conditions).	36
S	max_tempC_summer	Maximum summer temperature metric derived from a database containing seven years (2009–2015) of 30-min time series temperature logger data from 87 established sites the Calapooia basin. Maximum summer temperature was defined as the absolute maximum observation during the warmest period of the year in western Oregon, July–August. The maximum temperature value across all years considered was assigned to each site as a final representative maximum summer temperature metric.	36
S	amplitude	Amplitude and phase metrics for stream temperatures were calculated following the methods of Maheu et al. [42] to fit a sine curve to continuous time series data for each sample site and examine the magnitude and timing of temperature change throughout the year. This method provides a generalizable index of thermal regime magnitude (amplitude).	64
S	phase	Amplitude and phase metrics for stream temperatures were calculated following the methods of Maheu et al. [42] to fit a sine curve to continuous time series data for each sample site and examine the magnitude and timing of temperature change throughout the year. This method provides a generalizable index of thermal regime timing (phase).	64
S	v1w_msq	Large wood volumetric density (m^3^/m^2^), a measure of channel complexity and roughness. The physical habitat of stream segments was characterized using the methods of Kaufmann [39]. Data were collected 2013–2015 during the summer low-flow season.	19
S	sedembed	Sediment embeddedness (%), a measure of the degree to which substrate cobbles and gravels are encompassed by finer sediments. The physical habitat of stream segments was characterized using the methods of Kaufmann [39]. Data were collected 2013–2015 during the summer low-flow season.	20
S	sddepth	Morphology, using an index of variation in longitudinal variation in channel depth; a measure of pool/riffle ratio. The physical habitat of stream segments was characterized using the methods of Kaufmann [39]. Data were collected 2013–2015 during the summer low-flow season.	20
S	xfc_nat	In-channel cover (%), an index of channel complexity relevant to fish. The physical habitat of stream segments was characterized using the methods of Kaufmann [39]. Data were collected 2013–2015 during the summer low-flow season.	20
S	vegcovrip	Riparian vegetation cover (%), an index of riparian vegetation density and complexity. The physical habitat of stream segments was characterized using the methods of Kaufmann [39]. Data were collected 2013–2015 during the summer low-flow season.	20

		CHOP	

W	wetareasqm	Area of wetland polygons intersecting stream channels in a catchment in sq meters calculated using GIS tool ‘Tabulate by Intersection’ using NWI V2 and NHD v2 (and LiDAR) stream networks to quantify stream-wetland connectivity metrics.	523
W	wetpercentage	Percentage of areal coverage for wetland polygons intersecting stream channels in a catchment using GIS tool ‘Tabulate by Intersection’ using NWI V2 and NHD v2 (and LiDAR) stream networks to quantify stream-wetland connectivity metrics.	523
W	wetcntwhole	Count of whole wetland polygons in catchment calculated using GIS ‘Summarize FEATUREID on Spatial Join’ using NWI V2 and NHD v2 (and LiDAR) stream networks to quantify stream-wetland connectivity metrics.	523
W	wetcntpartial	Count of partial wetland polygons in catchment calculated using GIS ‘Summarize FEATUREID on Intersect’ using NWI V2 and NHD v2 (and LiDAR) stream networks to quantify stream-wetland connectivity metrics.	523
W	wetcntall	Count of total wetland polygons calculated as: Sum of wetcntwhole + wetcntpartial using NWI V2 and NHD v2 (and LiDAR) stream networks to quantify stream-wetland connectivity metrics.	523

		EFLMR	

S	log10Tavg	Log10 of the annual Total-N: Data collected between 2005 and 2015; Multiple site-analysis measurements within a day were averaged. Total Inorganic-N values greater than Total N values were removed from the analysis. Data were then organized by month between 2005–2015 and monthly average values were calculated for each site-analysis pairing.	43
S	log10TNH4aavg	Log10 of the annual total ammonium: Data collected between 2005 and 2015; Multiple site-analysis measurements within a day were averaged. Data were then organized by month between 2005–2015 and monthly average values were calculated for each site-analysis pairing.	43
S	log10TNOxaavg	Log10 of the annual total nitrate/nitrite: Data collected between 2005 and 2015; Multiple site-analysis measurements within a day were averaged. Data were then organized by month between 2005–2015 and monthly average values were calculated for each site-analysis pairing.	44
S	log10INavg	Data collected between 2005 and 2015; Multiple site-analysis measurements within a day were averaged. Data were then organized by month between 2005–2015 and monthly log10 of the average values were calculated for each site-analysis pairing.	44
S	log10fINavg	Fraction of Total-N as Inorganic-N: Data collected between 2005 and 2015; Multiple site-analysis measurements within a day were averaged. Data were then organized by month between 2005–2015 and monthly log10 of the average values were calculated for each site-analysis pairing.	38
S	log10TNdif	Annual range in Total-N: Monthly average values were used to compute the annual concentration fluctuation—Ndif, calculated as the difference between the highest and lowest monthly values of log10 Total-N concentrations.	43
S	log10TNH4dif	Annual range in total ammonium: Monthly average values were used to compute the annual concentration fluctuation—NH4dif, calculated as the difference between the highest and lowest monthly values of log10 total-ammonium concentrations.	43
S	log10TNOxdif	Annual range in total nitrate/nitrite: Monthly average values were used to compute the annual concentration fluctuation—NOxdif, calculated as the difference between the highest and lowest monthly values of log10 total nitrate/nitrite concentrations.	44
S	log10INdif	Annual range in Inorganic-N: Monthly average values were used to compute the annual concentration fluctuation—INdif, calculated as the difference between the highest and lowest monthly values of log10 Inorganic-N concentrations.	44
S	log10fINdif	Annual range in fraction of Total-N as Inorganic-N: Monthly average values were used to compute the annual concentration fluctuation—fINdif, calculated as the difference between the highest and lowest monthly values of fraction of log10 Total-N as Inorganic-N concentrations.	38

		NBW	

S	δ^15^N periphyton	Nitrogen isotopic composition (δ^15^N‰) of periphyton collected from six randomly selected rocks (composite sample) at stream sites within Narragansett Bay Watershed in 2012	69
S	log10tn	Total nitrogen log 10 transformed-water sample collected from stream sites in 2012	71
S	log10no3	NO_3_ concentration log 10 transformed-water sample collected from stream sites in 2012	71
S	log10nh4	NH_4_ concentration log 10 transformed-water sample collected from stream sites in 2012	71
S	log10chloride	Chloride concentration log 10 transformed- water sample collected from stream sites in 2012	71
L	δ^15^N BOM	Nitrogen isotopic composition (δ^15^N‰) of benthic organic matter (BOM) collected from surficial sediments in littoral zone of lakes	51

CRW = Calapooia River Watershed; CHOP = Choptank Study Area Watershed; EFLMR = East Fork Little Miami River; NBW = Narragansett Bay Watershed. Type: S = stream; W = wetland; L = lake.

**Table 6:** Summary statistics (minimum, 25th percentile, mean, median, 75th percentile, maximum and standard deviation) for the Indices of Watershed Integrity and Catchment Integrity for sampled sites, and summary statistics for all sites (not just sampled sites) within each of the four case study watersheds.

Case Study Watershed	Index of Watershed Integrity							Index of Catchment Integrity						

	min	25%	mean	median	75%	max	SD	min	25%	mean	median	75%	max	SD
CRW	0.418	0.642	0.789	0.876	0.962	0.982	0.195	0.423	0.495	0.732	0.75	0.959	0.983	0.225
CHOP	0.403	0.511	0.556	0.55	0.594	0.822	0.069	0.386	0.509	0.601	0.578	0.662	0.957	0.128
EFLMR	0.409	0.456	0.546	0.494	0.638	0.918	0.117	0.403	0.513	0.62	0.592	0.754	0.923	0.138
NBW	0.427	0.674	0.746	0.764	0.837	0.923	0.113	0.418	0.675	0.75	0.754	0.865	0.925	0.122
Watersheds Combined Sampled Sites	0.403	0.52	0.641	0.588	0.747	0.982	0.162	0.386	0.518	0.659	0.614	0.793	0.983	0.171
Watersheds Combined All Sites a	0.19	0.562	0.68	0.691	0.796	1	0.146	0.125	0.571	0.7	0.71	0.831	1	0.157

CRW = Calapooia River Watershed; CHOP = Choptank Study Area Watershed; EFLMR = East Fork Little Miami River; NBW = Narragansett Bay Watershed.

a All sites: summary statistics for all sites-not just sampled sites within each watershed.

**Table 7:** Pearson correlations for Calapooia River Watershed response metrics with Indices of Watershed (IWI) and Catchment Integrity (ICI) and associated six functional component indices.

Index	Vegcovrip	TN_in	Sedembed	Phase	Max_tempC_Summer	log10Ndif	log10NAavg	Fish MMI	δ^15^N Chironomid
IWI	0.65	− 0.97	−0.77	0.77	− 0.73	− 0.93	− 0.92	0.82	− 0.92
ICI	0.52	− 0.96	− 0.75	0.76	− 0.91	− 0.82	− 0.77	0.83	− 0.89
WCHEM	0.63	− 0.99	− 0.74	0.77	− 0.70	− 0.91	− 0.91	0.79	− 0.88
CCHEM	0.49	− 0.98	− 0.74	0.74	− 0.89	− 0.84	− 0.79	0.81	− 0.87
WHABT	0.59	− 0.97	− 0.71	0.75	− 0.68	− 0.90	− 0.90	0.78	− 0.87
CHABT	0.48	− 0.97	− 0.72	0.75	− 0.88	− 0.82	− 0.76	0.80	− 0.86
WSED	0.57	− 0.98	− 0.69	0.75	− 0.67	− 0.89	− 0.91	0.73	− 0.82
CSED	0.45	− 0.98	− 0.68	0.76	− 0.87	− 0.84	− 0.80	0.77	− 0.83
WHYD	0.63	− 0.99	− 0.74	0.77	− 0.71	− 0.90	− 0.90	0.78	− 0.88
CHYD	0.49	− 0.98	− 0.75	0.73	− 0.87	− 0.83	− 0.78	0.80	− 0.86
WTEMP	0.63	− 0.98	− 0.74	0.77	− 0.71	− 0.90	− 0.90	0.79	− 0.88
CTEMP	0.48	− 0.98	− 0.73	0.75	− 0.90	− 0.82	− 0.76	0.81	− 0.86
WCONN	0.72	− 0.94	− 0.82	0.71	− 0.65	− 0.92	− 0.91	0.84	− 0.93
CCONN	0.58	− 0.92	− 0.78	0.74	− 0.87	− 0.70	− 0.66	0.83	− 0.87

See key for response metric descriptions below. All entries significant at *p* < 0.001.

Vegcovrip: riparian vegetation cover (%), an index of riparian vegetation density and complexity; TN_in: annual total nitrogen (TN) input from all anthropogenic and natural sources; Sedembed: sediment embeddedness (%), a measure of the degree to which substrate cobbles and gravels are encompassed by finer sediments; Phase: generalizable index of stream thermal regime timing; Max temp_C_summer: maximum summer temperature defined as the absolute maximum observation during the warmest period of the year in western Oregon, July–August; log10Ndif: annual fluctuation calculated as the difference between the highest and lowest monthly values of log10 NO_3_ concentrations; log10Naavg: average log10 NO_3_ concentrations of each month over the sampling period; fish MMI: Fish multimetric indices; δ^15^N Chironomid: chironomid nitrogen isotopic composition (δ^15^N ‰) were collected from 2013–2015.

**Table 8:** Spearman rank correlations for the Choptank Watershed study area response metrics with Indices of Watershed (IWI) and Catchment Integrity (ICI) and associated six functional component indices using NHDPlusV2 methods for stream network characterization.

Index	Wetareasqm	Wetpercentage	Wetcntall	Wetcntwhole	Wetcntpartial
IWI	0.36 ***	0.30 ***	0.24 ***	0.10 *	0.33 ***
ICI	0.19 ***	0.49 ***	0.01 −	− 0.11 **	0.20 ***
WHYD	0.35 ***	0.29 ***	0.25 ***	0.11 *	0.32 ***
CHYD	0.16 **	0.46 ***	0.03−	− 0.07−	0.19 ***
WCHEM	0.34 ***	0.28 ***	0.25 ***	0.10 *	0.32 ***
CCHEM	0.16 **	0.47 ***	0.02−	− 0.09−	0.19 ***
WSED	0.32 ***	0.26 ***	0.27 ***	0.13 **	0.33 ***
CSED	0.13 **	0.45 ***	0.01−	− 0.12 *	0.19 ***
WCONN	0.26 ***	0.21 ***	0.13 **	0.06−	0.16 **
CCONN	0.14 **	0.41 ***	−0.10 *	− 0.19 ***	0.14 **
WTEMP	0.35 ***	0.30 ***	0.23 ***	0.09−	0.32 ***
CTEMP	0.16 **	0.46 ***	0.01−	− 0.09−	0.18 ***
WHABT	0.39 ***	0.34 ***	0.23 ***	0.07−	0.34 ***
CHABT	0.18 ***	0.49 ***	0.01−	−0.11 *	0.19 ***

See key for response metric descriptions below. Significance levels:

*** *p* < 0.001;

** *p* < 0.01;

* *p* < 0.05;

− *p* < 0.1; blank signifies model not significant.

Wetareasqm: area of wetland polygons intersecting stream channels in a catchment in sq meters; Wetpercentage: percentage of areal coverage for wetland polygons intersecting stream channels in a catchment; Wetcntall: count of total wetland polygons calculated as: sum of wetcntwhole + wetcntpartial; Wetcntwhole: count of whole wetland polygons in catchment; Wetcntpartial: count of partial wetland polygons in catchment.

**Table 9:** Pearson correlations for the East Fork Little Miami Watershed response metrics with Indices of Watershed (IWI) and Catchment Integrity (ICI) and associated six functional component indices.

Index	log10T avg	log10TNH4aavg	log10TNOxaavg	log10INavg	log10fINavg	log10TNdif	log10TNH4dif	log10TNOxdif	log10INdif	log10fINdif
IWI	−0.78 ***	−0.44 ***	−0.64 ***	− 0.64 ***	0.01	− 0.61 ***	− 0.27 ***	− 0.76 ***	− 0.65 ***	− 0.1
ICI	− 0.56 ***	− 0.51 ***	− 0.46 ***	− 0.5 ***	0.05	− 0.54 ***	− 0.45 ***	− 0.61 ***	− 0.58 ***	− 0.34 ***
WCHEM	− 0.72 ***	− 0.4 ***	− 0.63 ***	− 0.62 ***	− 0.03	− 0.56 ***	− 0.25 ***	− 0.74 ***	− 0.62 ***	− 0.11 **
CCHEM	− 0.53 ***	− 0.46 ***	− 0.49 ***	− 0.52 ***	− 0.03	− 0.51 ***	− 0.4 ***	− 0.6 ***	− 0.56 ***	− 0.33 ***
WHABT	− 0.8 ***	− 0.48 ***	− 0.65 ***	− 0.66 ***	0.01	− 0.64 ***	− 0.3 ***	− 0.77 ***	− 0.67 ***	− 0.13 **
CHABT	− 0.56 ***	− 0.49 ***	− 0.46 ***	− 0.5 ***	0.06	− 0.56 ***	− 0.43 ***	− 0.62 ***	− 0.6 ***	− 0.39 ***
WSED	− 0.8 ***	− 0.49 ***	− 0.59 ***	− 0.61 ***	0.08	− 0.68 ***	− 0.33 ***	− 0.77 ***	− 0.69 ***	− 0.13 **
CSED	− 0.62 ***	− 0.52 ***	− 0.48 ***	− 0.53 ***	0.09	− 0.66 ***	− 0.45 ***	− 0.68 ***	− 0.67 ***	− 0.44 ***
WHYD	− 0.8 ***	− 0.47 ***	− 0.59 ***	− 0.62 ***	0.11	− 0.69 ***	− 0.31 ***	− 0.77 ***	− 0.72 ***	− 0.14 **
CHYD	− 0.57 ***	− 0.48 ***	− 0.44 ***	− 0.49 ***	0.16	− 0.64 ***	− 0.44 ***	− 0.65 ***	− 0.65 ***	− 0.43 ***
WTEMP	− 0.78 ***	− 0.45 ***	− 0.63 ***	− 0.64 ***	0.03	− 0.61 ***	− 0.28 ***	− 0.74 ***	− 0.65 ***	− 0.1 **
CTEMP	− 0.51 ***	− 0.52 ***	− 0.41 ***	− 0.46 ***	0.06	− 0.5 ***	− 0.48 ***	− 0.55 ***	− 0.55 ***	− 0.31 ***
WCONN	− 0.77 ***	− 0.52 ***	− 0.6 ***	− 0.64 ***	0.02	− 0.65 ***	− 0.36 ***	− 0.74 ***	− 0.69 ***	− 0.13 **
CCONN	− 0.45 ***	− 0.52 ***	− 0.32 ***	− 0.39 ***	0.05	− 0.37 ***	− 0.47 ***	− 0.41 ***	− 0.44 ***	− 0.06 −

See key for response metric descriptions below. Significance levels:

*** *p* < 0.001;

** *p* < 0.01;

* *p* < 0.05;

− *p* < 0.1; blank signifies model not significant.

log10Tavg: annual log10 Total-N: collected between 2005 and 2015; log10TNH4aavg: annual log10 total ammonium: collected between 2005 and 2015; log10TNOxaavg: annual log10 total nitrate/nitrite: collected between 2005 and 2015; log10INavg: Inorganic-log10 N: Data collected between 2005 and 2015; log10fINavg: fraction of Total-N as Inorganic-log10 N: Data collected between 2005 and 2015; log10TNdif: Annual range in log10 Total-N; log10TNH4dif: Annual range in log10 total ammonium; log10TNOxdif: Annual range in log10 total nitrate/nitrite; log10INdif: Annual range in log10 Inorganic-N; log10fINdif: Annual range in fraction of Total-N as log10 Inorganic-N.

**Table 10:** Pearson correlations for the Narragansett Bay Watershed response metrics with Indices of Watershed (IWI) and Catchment Integrity (ICI) and associated six functional component indices.

Index	δ^15^N Periphyton	Log10tn	Log10no3	Log10nh4	Log10chloride	δ^15^N BOM
IWI	−0.47 ***	−0.48 ***	−0.52 ***	− 0.31 ***	−0.57 ***	−0.64 ***
ICI	− 0.35 ***	− 0.31 ***	− 0.6 ***	− 0.23 ***	− 0.68 ***	− 0.58 ***
WCHEM	− 0.44 ***	− 0.43 ***	− 0.54 ***	− 0.34 ***	− 0.6 ***	− 0.62 ***
CCHEM	− 0.3 ***	− 0.26 ***	− 0.47 ***	− 0.2 ***	− 0.5 ***	− 0.61 ***
WHABT	− 0.41 ***	− 0.46 ***	− 0.53 ***	− 0.28 ***	− 0.59 ***	− 0.51 ***
CHABT	− 0.25 ***	− 0.23 ***	− 0.41 ***	− 0.18 ***	− 0.46 ***	− 0.61 ***
WSED	− 0.49 ***	− 0.48 ***	− 0.59 ***	− 0.31 ***	− 0.69 ***	− 0.55 ***
CSED	− 0.27 ***	− 0.25 ***	− 0.47 ***	− 0.15 **	− 0.55 ***	− 0.63 ***
WHYD	− 0.55 ***	− 0.43 ***	− 0.52 ***	− 0.31 ***	− 0.62 ***	− 0.51 ***
CHYD	− 0.41 ***	− 0.32 ***	− 0.48 ***	− 0.19 ***	− 0.56 ***	− 0.59 ***
WTEMP	− 0.43 ***	− 0.46 ***	− 0.58 ***	− 0.3 ***	− 0.63 ***	− 0.51 ***
CTEMP	− 0.34 ***	− 0.31 ***	− 0.49 ***	− 0.23 ***	− 0.5 ***	− 0.56 ***
WCONN	− 0.35 ***	− 0.42 ***	− 0.56 ***	− 0.23 ***	− 0.63 ***	− 0.49 ***
CCONN	− 0.32 ***	− 0.27 ***	− 0.46 ***	− 0.21 ***	− 0.48 ***	− 0.54 ***

See key for response metric descriptions below. Significance levels:

*** *p* < 0.001;

** *p* < 0.01;

* *p* < 0.05;

− *p* < 0.1; blank signifies model not significant.

δ^15^N periphyton: nitrogen isotopic composition (δ^15^N ‰) of periphyton collected from stream site in 2012; log10tn: total nitrogen log 10 transformed-water sample collected from stream site in 2012; log10no3: NO_3_ (nitrate) concentration log 10 transformed-water sample collected from stream site in 2012; log10nh4: NH_4_ (ammonia) concentration log 10 transformed-water sample collected from stream site in 2012; log10chloride: chloride concentration log 10 transformed-water sample collected from stream site in 2012; δ^15^N BOM: nitrogen isotopic composition (δ^15^N ‰) of benthic organic matter (BOM) collected from surficial sediments in littoral zone of lake.

**Table 11:** Pearson correlations for case study response variables with individual landscape variables percent urban (%Urb), percent forest (%For) and percent agriculture (%Agr) at watershed and catchment scales, compared with the Index of Watershed Integrity (IWI) or Index of Catchment Integrity (ICI). Spearman’s rank correlation coefficient was used as a non-parametric rank statistic in the Choptank watershed because response variables were not normally distributed. Maximum absolute correlations for each response variable and scale indicated in bold.

Response Variable	WATERSHED SCALE							CATCHMENT SCALE						

	% Urb	% For	% Agr	IWI	% Max	Rank	# Exceed	% Urb	% For	% Agr	ICI	% Max	Rank	#Exceed
**CRW**														

δ^15^N chironomid	0.45	−0.79	0.83	**−0.92**	100.0	1	3	0.12	−0.86	0.83	**−0.89**	100.0	1	3
total in	0.92	−0.87	**0.98**	−0.97	99.0	2	2	0.85	−0.86	**0.98**	−0.96	98.0	2	2
Log10Ndif	0.59	−0.9	0.9	**−0.93**	100.0	1	3	0.3	−0.78	0.8	**−0.82**	100.0	1	3

**EFLMR**														

log10Tavg	−0.56	−0.72	**0.8**	−0.78	97.5	2	2	0	**− 0.57**	0.54	−0.56	98.2	2	2
10g10TNH_4_aavg	−0.39	−0.39	**0.49**	−0.44	89.8	2	2	−0.07	−0.43	0.47	**−0.51**	100.0	1	3
10g10TNOxaavg	−0.28	−0.63	0.57	**−0.64**	100.0	1	3	0.14	**− 0.53**	0.4	−0.46	86.8	2	2
10g10DIavg	−0.37	−0.61	0.61	**−0.64**	100.0	1	3	0.12	**− 0.55**	0.44	−0.5	90.9	2	2
10g10fDIavg	**0.3**	−0.06	−0.17	0.01	3.3	4	0	**0.43**	−0.06	−0.22	0.05	11.6	4	0
10g10TNdif	−0.61	−0.53	**0.72**	−0.61	84.7	2.5	1	−0.29	−0.49	**0.65**	−0.54	83.1	2	2
10g10TNH_4_dif	−0.31	−0.24	**0.35**	−0.27	77.1	3	1	−0.18	−0.34	**0.45**	**− 0.45**	100.0	1.5	2
10g10TNOxdif	−0.49	−0.72	**0.76**	**− 0.76**	100.0	1.5	2	−0.1	−0.62	**0.63**	−0.61	96.8	3	1
10g10DINdif	−0.59	−0.59	**0.74**	−0.65	87.8	2	2	−0.18	−0.56	**0.64**	−0.58	90.6	2	2
10g10fDINdif	−0.15	−0.11	**0.16**	−0.1	62.5	4	0	−0.28	−0.26	**0.45**	−0.34	75.6	2	2

**NBW**														

δ^15^N periphyton	0.45	**−0.5**	0.11	−0.47	94.0	2	2	0.39	**− 0.48**	0.07	−0.35	72.9	3	1
10g10tn	0.49	**−0.52**	−0.02	−0.48	92.3	3	1	0.33	**− 0.43**	0.02	−0.31	72.1	3	1
10g10NO_3_	0.55	−0.47	−0.07	**−0.6**	100.0	1	3	**0.52**	−0.51	−0.07	**−0.52**	100.0	1.5	2
10g10NH_4_	0.27	**−0.37**	−0.08	−0.31	83.8	2	2	0.22	**− 0.33**	−0.09	−0.23	69.7	2	2
10g10ch10ride	0.59	−0.63	−0.04	**−0.68**	100.0	1	3	0.56	**− 0.62**	−0.02	−0.57	91.9	2	2
δ^15^N BOM	0.66	**−0.68**	0.14	−0.58	85.3	3	1	0.64	**− 0.68**	0.13	−0.64	94.1	2.5	1

**CHOP**														

wetpercentage	−0.08	0.23	−0.29	**0.3**	100.0	1	3	−0.18	0.22	−0.46	**0.49**	100.0	1	3
wetcntall	−0.08	0.21	**−0.24**	**0.24**	100.0	1.5	2	0.3	**0.35**	−0.04	0.01	2.9	4	0
wetcntwhole	−0.02	0.07	**−0.1**	**0.1**	100.0	1.5	2	**0.37**	0.27	0.07	−0.11	29.7	3	1
wetcntpartial	−0.11	0.29	−0.31	**0.33**	100.0	1	3	0.06	**0.29**	−0.19	0.2	69.0	2	2

CRW = Calapooia River Watershed; EFLMR = East Fork Little Miami River; NBW = Narragansett Bay Watershed; CHOP = Choptank Watershed study area; %Max = IWI or ICI absolute correlation value divided by the maximum absolute correlation value for each response variable and scale; Rank = rank of the IWI or ICI absolute correlation value for each response variable and scale; #Exceed = the number of individual landscape variables with an absolute correlation less than the IWI or ICI absolute correlation for each response variable and scale. See Table 4 for description of response variables.
