# Supplementary material for: Performance of National Maps of Watershed Integrity at Watershed Scales
Source: Water (Basel). Author manuscript; Available in PMC 2018 Aug 2. (PMC6071426; doi:10.3390/w10050604)
Supplement: Supplement1 [file NIHMS971798-supplement-Supplement1.pdf]

water

MDPI

Supplementary Materials: The following are available online at www.mdpi.com/link.

Table S1. Pearson correlations for Calapooia River Watershed nitrogen budget indices with Indices of Watershed (IWI) and Catchment Integrity (ICI). Indices were calculated in the Calapooia River Watershed N budget project [1], which quantified total nitrogen (TN) input, export, and retention in Calapooia. See key for response metric descriptions below. All entries significant at $p < 0.001$.

Index	harvest_rmvl	res_N	total_out	Aag_frt_in	winter_frt
IWI	−0.91	−0.96	−0.88	−0.97	−0.96
ICI	−0.9	−0.95	−0.88	−0.96	−0.95
WCHEM	−0.94	−0.95	−0.91	−0.99	−0.98
CCHEM	−0.94	−0.94	−0.92	−0.98	−0.98
WHABT	−0.91	−0.96	−0.88	−0.97	−0.96
CHABT	−0.91	−0.95	−0.89	−0.97	−0.96
WSED	−0.94	−0.93	−0.92	−0.98	−0.98
CSED	−0.94	−0.92	−0.93	−0.98	−0.98
WHYD	−0.93	−0.95	−0.91	−0.99	−0.98
CHYD	−0.93	−0.94	−0.91	−0.98	−0.98
WTEMP	−0.93	−0.95	−0.91	−0.99	−0.97
CTEMP	−0.93	−0.94	−0.91	−0.98	−0.97
WCONN	−0.87	−0.95	−0.84	−0.94	−0.92
CCONN	−0.85	−0.94	−0.82	−0.93	−0.9

Note: harvest (kg N/ha/yr): Annual N removal via crop harvest (Note: Harvest is based on high resolution agricultural land use and so may be circular with respect to variables within the IWI and ICI); resN (kg N/ha/yr): Retention N—difference between annual TN input and annual TN export; total_out (kg N/ha/yr): Annual total nitrogen (TN) export using LOADEST model [1]; Aag_frt (kg N/ha/yr): Annual TN input from fertilization; winter_frt (kg N/ha): fertilizer N input in winter

Table S2. Spearman rank correlations for the Choptank River Watershed study area response metrics and indices with Indices of Watershed (IWI) and Catchment Integrity (ICI) using LiDAR for stream network characterization. See key for response metric descriptions below. Significance levels: '***' $p < 0.001$; '**' $p < 0.01$; '*' $p < 0.05$; '- '$p < 0.1$; blank signifies model not significant.

**index**	**wetareasqm**	**wetpercentage**	**wetcntall**	**wetcntwhole**	**wetcntpartial**
IWI	0.43***	0.42***	0.31***	0.20***	0.41***
ICI	0.19***	0.56***	0.04-	−0.04	0.22***
WHYD	0.42***	0.43***	0.32***	0.22***	0.42***
CHYD	0.18***	0.56***	0.07-	0.00-	0.23***
WCHEM	0.41***	0.41***	0.31***	0.21***	0.42***
CCHEM	0.19***	0.57***	0.05-	−0.02	0.22***
WSED	0.39***	0.38***	0.30***	0.20***	0.41***
CSED	0.16**	0.56***	0.02-	−0.06-	0.21***
WCONN	0.25***	0.23***	0.14**	0.09-	0.18***

CCONN	0.04^{-}	0.34^{***}	-0.12^{***}	-0.16^{**}	0.04^{-}
WTEMP	0.42^{***}	0.43^{***}	0.30^{***}	0.20^{***}	0.41^{***}
CTEMP	0.19^{***}	0.56^{***}	0.06^{-}	-0.01^{-}	0.22^{***}
WHABT	0.46^{***}	0.47^{***}	0.31^{***}	0.20^{***}	0.43^{***}
CHABT	0.21^{***}	0.61^{***}	0.06^{-}	-0.02^{-}	0.24^{***}

wetareasqm: area of wetland in the catchment in square meters; wetpercentage: percentage of areal coverage for wetland polygons intersecting stream channels in a catchment; wetcntall: count of total wetland polygons in a catchment, includes whole and partial wetlands within catchment; wetcntwhole: count of whole wetland polygons in catchment; wetcntpartial: count of partial wetland polygons in catchment.

Detailed Methods for Response Variables

Calapooia River Watershed (CRW)

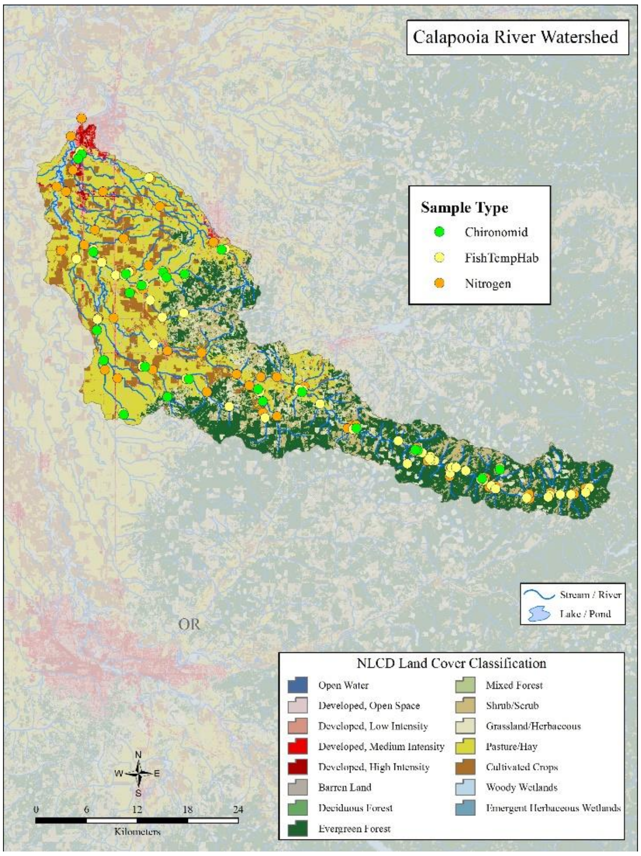

Figure S1. Stream sample site locations in the Calapooia River Watershed

Annual nitrate concentration fluctuation index (Ndif)

One of the nitrogen-related metrics included a measure of the annual range in average nitrate concentration from surface water samples collected at over 70 stream sampling sites that were selected to cover the range of land uses in the CRW (Figure S1). Of the 73 sampled stream site locations, 53 stream sites were associated with NHDPlusV2 flowlines and linked to their respective IWI/ICI values along with their associated six individual functional components. Stream sites were sampled intermittently from 2003–2006 by the US Department of Agriculture-Agricultural Research Service (USDA-ARS) and Oregon State University (monthly or quarterly), and from 2009–2011 by US EPA (quarterly). Nitrate-N (mg/L) was analyzed using a Lachat Quick Chem 4200 analyzer [1,2] At each sampled stream site, average NO_3^- concentrations for each month over the entire sampling

period were calculated. For example, at a given site, if water samples were taken from 2004 to 2006, monthly average nitrate concentration (N_i, in mg/L) is calculated as:

$$N_i = \frac{1}{3}\left(N_{i,2004} + N_{i,2005} + N_{i,2006}\right)(i = [Jan, Dec]) \quad (1)$$

where, i represents each of the twelve months of a year. These monthly average values were then used to compute the annual concentration fluctuation (N_{dif}), calculated as:

$$N_{dif} = N_{max} - N_{min} \quad (2)$$

where, N_{max} and N_{min} are respectively the highest and lowest monthly averages. The log10 transformed N_{dif} is the final selected NO_3^- concentration fluctuation index that was used as the response metric to compare with IWI/ICI values and six associated functional components.

Watershed nitrogen budget index: annual total nitrogen input

Indices were calculated as part of a CRW N budget project [1], which quantified total nitrogen (TN) input at the same 53 stream sampling sites in the CRW described above. Seven sources of TN input were estimated: land application of agricultural fertilizer, manure from Concentrated Animal Feeding Operations (CAFOs), atmospheric deposition, biological N fixation (BNF) by crops, BNF associated with red alder (*Alnus rubra*) trees, non-agricultural fertilizer applied to developed lands, and non-sewered septic waste. Total nitrogen input was calculated from these seven sources at mainstem and tributary level watersheds and subwatershed levels. ArcGIS layers of nitrogen sources were clipped to these boundaries, and utilized to quantify contributions of various N sources in the study area [1]. Annual TN input (*Total_in* (kg-N ha^{-1} yr^{-1})) to the CRW from all anthropogenic and natural sources at 54 stream sites was used as a watershed scale response metric to compare with the respective IWI/ICI values and six associated functional components.

Stable isotope ratios of $\delta^{15}N$ and $\delta^{13}C$ for chironomids

Measuring nitrogen stable isotope ratios ($^{15}N/^{14}N$ expressed as $\delta^{15}N$) in organisms has become a useful approach for determining effects of different nitrogen sources on aquatic ecosystems [3]. In general, $\delta^{15}N$ values in macroinvertebrates increase with watershed-level agricultural land cover and N loading, and can be used as indicators of both nitrogen sources and nitrogen removal processes within lakes, rivers and streams. Stable isotopes of $\delta^{13}C$ and $\delta^{15}N$ were measured from chironomids collected at 31 stream site locations within the CRW from 2013–2015 (Figure S1). Chironomids were collected in the field following National River and Stream Assessment (NRSA) methods [4]. EcoAnalysts, Inc. (Moscow, Idaho) performed taxonomic identification of chironomids from the benthic macroinvertebrate samples. Both $\delta^{13}C$ and $\delta^{15}N$ were measured at the US EPA Western Ecology Division's Integrated Stable Isotope Research Facility in Corvallis, Oregon. Stable isotope ratios of samples were determined using a continuous flow isotope ratio mass spectrometer (Isoprime 100 Mass Spectrometer, Elementar Americas, Mt. Laurel, NJ, USA) connected to an elemental analyzer (Vario Isotope Cube, Elementar Americas) and reported as parts per thousand differences between samples and the reference standard ($\delta^{15}N$ ‰ and $\delta^{13}C$ ‰). Of the 31 sampled stream sites, 22 were associated with NHDPlusV2 flowlines and were linked to their respective IWI/ICI values along with their associated six individual functional components.

Stream sampling for fish assemblages, chemistry, temperature and physical habitat

In the Calapooia, sites were chosen to capture broad gradients in physical setting, stream size, and watershed condition (Figure S1). Sampling sites were almost entirely located on privately-owned residential, agricultural, or commercial forestlands which make of the vast majority of the stream network length. Sampling site locations were severely constrained by landowner permissions. At each site sampled for fish assemblages, we collected water chemistry, temperature, and physical habitat information, unless landowners specifically requested that we abstain from

doing so. The two dams in the basin were removed in 2008 and 2011. We did not sample within 1,000m above or below existing or former dam sites. We worked closely with the local watershed organization, the Calapooia Watershed Council, to select sites for fish, water quality, physical habitat and water temperature sampling based on identified restoration needs of the Council, gaps in existing knowledge, and landowner cooperation. For example, some sites were located upstream and downstream of stream sections targeted for riparian restoration efforts over the next 10 years, to set baseline conditions for monitoring purposes. Other sites were located in parts of the basin that had not been previously sampled by state agencies, and where information on status of habitat and fish assemblage condition was desired by local landowners and restoration planners working with the Watershed Council. We used Spatial Stream Network (SSN) models to predict temperatures at un-monitored portions of the network, and to identify priority areas (based on high standard errors of predictions) for addition of sampling sites and we were able to reduce some uncertainty in this manner. But we only used observed (not predicted) temperatures for this analysis; the SSN modeling helps give us confidence about our spatial representation.

Stream physical habitat

The physical habitat of stream segments was characterized using the methods of Kaufmann (2006) [5]. Physical habitat data were collected from 2013–2015 for 20 streams within the CRW during the summer low-flow season (Figure S1). The following two stream physical habitat metrics were extracted for comparison and analysis to capture functional processes of interest that had been previously identified by Kaufmann [5, 6 (see Table 19)] as among those most reliable and commonly used: sediment embeddedness (%, *sedembed*), a measure of the degree to which substrate cobbles and gravels are encompassed by finer sediments; and riparian vegetation cover (%, *vegcovrip*), an index of riparian vegetation density and complexity. These two measures of physical habitat for 20 streams within the CRW were used as response variables to compare with IWI/ICI values and their six associated functional components.

Stream temperature

Stream water temperature was measured using temperature loggers (Optic TidBits model TBI32; Onset Computer Corp., Pocasset, Massachusetts) placed in a well-mixed portion of the stream channel following methods of Dunham *et al*. (2005) [7]. The maximum summer temperature metric was derived from a database containing seven years (2009 – 2015) of 30-minute time series temperature logger data from 87 established sites within the Calapooia basin (Figure S1). Maximum summer temperature was defined as the absolute maximum observation during the warmest period of the year in western Oregon, July – August. Raw data were processed using R statistical software [8] to screen for completeness and remove observations where loggers were exposed to air. Any sites containing less than 90% continuous summer time coverage in a given year were excluded from further analysis. The maximum temperature value across all years considered was assigned to each site as a final representative maximum summer temperature metric. Forty-five of the 87 sites sampled met the minimum observation threshold for at least one year of data, 36 of which corresponded to NHD PlusV2 stream lines and were used for further comparison with the IWI/ICI values and six associated functional components.

Amplitude and phase

Amplitude and phase metrics for stream temperatures were calculated following the methods of Maheu *et al*. (2015) [9] in order to fit a sine curve to continuous time series data for each sample site and examine the magnitude and timing of temperature change throughout the year. This method provides a generalizable index of thermal regime magnitude (amplitude) and timing (phase). The index of thermal regime timing, *Phase*, was calculated for 64 stream site locations within the CRW, and these values were used as a timing response variable to compare with IWI/ICI values and six associated functional components.

Fish multimetric index (Fish MMI)

Fish sampling data used to derive multimetric indices (MMIs) in the Calapooia basin were collected between 2010 and 2014 using methodologies consistent with those used by US EPA's Environmental Monitoring and Assessment Program (EMAP) [10] and NRSA [4]. In total, 50 sites were visited over four years (no sampling occurred in 2012) and ranged from forested headwater streams in the Cascade mountains to lowland reaches in high density agricultural areas of the Willamette Valley. Study reaches were 40 times the active channel width and were sampled using a single pass with backpack electrofisher [11]. Sampling occurred throughout the year but for consistency, only data collected during the NRSA index period (June through September) were used for this analysis (summer low-flow conditions). MMIs were calculated following methods set by Whittier *et al*. (2007) [12]. Seven metrics for the Western Mountains Ecoregion were selected and consisted of an assemblage tolerance index estimating overall resilience to disturbance [12], along with proportional measures of sensitive rheophilic species, sensitive invertivores-piscivores, lithophilic spawners, salmonid abundance, native sensitive long-lived species, and alien vertebrate presence. Scores were generated for each unique sampling event and a mean inter-annual score was attributed to sites receiving multiple visits over the course of the study. Final scores were rescaled to values between 0 and 100 for comparison with the IWI/ICI values, with increasing scores indicating higher overall ecological condition.

East Fork Little Miami River (EFLMR) Watershed

Data were organized by month between 2005–2015 and monthly average values were calculated for each site-analysis pairing, following Eq. 1 (see *Annual nitrate concentration fluctuation index (Ndif) section under* Calapooia River Watershed methods above). However, the number of years of data varied for each EFLMR site. Monthly averages for site-analysis pairings with less than 3 observations for a given month were left blank. Annual averages were calculated by averaging the 12 monthly average values for each site-analysis pairing. Sites with data for less than 10 separate months were removed from further analysis. This left 44 sites that could be associated with IWI/ICI values for further analysis (Figure S2). Annual range values were calculated using the monthly summaries, following Eq. 2 (see *Annual nitrate concentration fluctuation index (Ndif) section under* Calapooia River Watershed methods above). Annual mean values (N_{mean}, in μg/L) were also calculated in a similar fashion:

$$\boldsymbol{N_{mean}} = \frac{1}{n}\sum_{i=1}^{n}\boldsymbol{N_i}, \tag{3}$$

where N_i is the monthly average. Annual range and mean values were then log10 transformed.

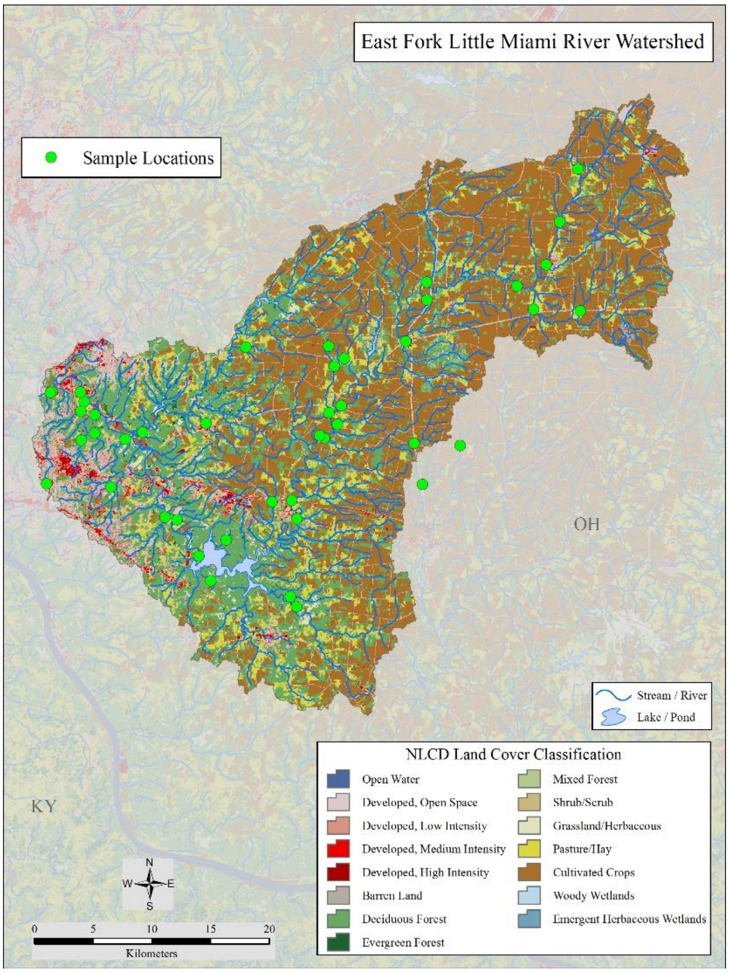

Figure S2. Stream sample site locations in the East Fork Little Miami River Watershed

Narragansett Bay Watershed (NBW)

Stable isotope ratios of δ^{15}N and δ^{13}C for periphyton

Stable isotope ratios of δ^{15}N and δ^{13}C of periphyton (or biofilm, which consists of microbial communities, predominantly bacteria, encased in a layer of extracellular polymeric substances) have been shown to be effective indicators of watershed development effects on stream ecosystems [3, 13]. This can also make them useful for quantifying the effectiveness of nitrogen, stream, and watershed management efforts [3]. Periphyton was simultaneously collected at the same 77 stream sites within NBW where single grab sample surface water chemistry was collected between July and October 2012 (Figure S3) [14]. Six stones evenly dispersed throughout a 50 m stream reach encompassing a riffle were randomly collected in a zigzag pattern, and periphyton was removed from their surfaces using a firm bristled brush [14]. Periphyton samples were stored in the dark on ice until returning to the lab where they were processed within 24 hours. For each sample, a 50 ml subsample of homogenized periphyton was concentrated into a pellet using a centrifuge and then dried, weighed, and analyzed for δ^{15}N and δ^{13}C stable isotope ratios. Stable isotope ratios of samples were determined using a continuous flow isotope ratio mass spectrometer (Isoprime 100 Mass Spectrometer, Elementar Americas, Mt. Laurel, NJ, USA) and reported as per mil differences (‰) between samples and reference materials (δ^{15}N and δ^{13}C). Atmospheric N_2 and Pee Dee Belemnite were reference standards for nitrogen and carbon stable isotopes, respectively [15, 16].

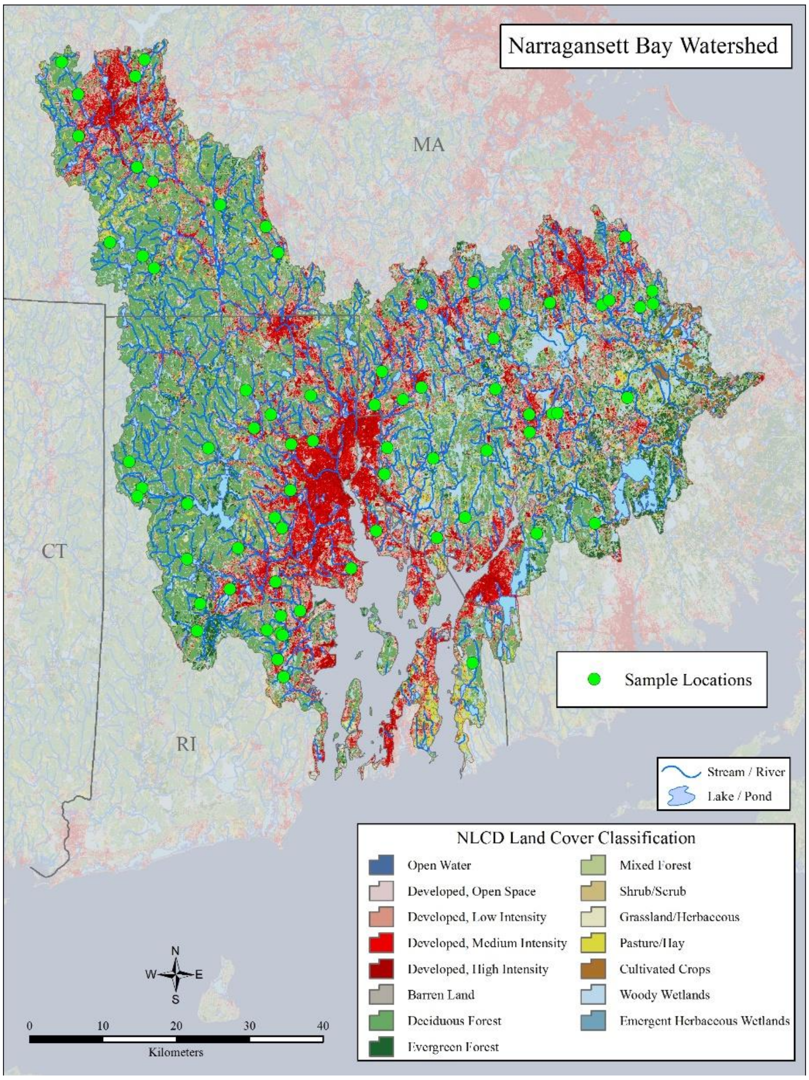

Figure S3. Stream sample site locations in the Narragansett Bay Watershed

Stable isotope ratios of $\delta^{15}N$ and $\delta^{13}C$ for benthic organic matter in lakes

Increases in stable isotope ratios of $\delta^{15}N$ and $\delta^{13}C$ of benthic organic matter (BOM) collected from surficial sediments in lakes are associated with increases in impervious surface and population density and decreases in forested land in watershed and buffer zones surrounding lakes [15]. We used stable isotope ratios collected from BOM samples in lakes to evaluate how these measures of aquatic condition are correlated with IWI/ICI values. Samples of benthic organic matter were collected from the littoral zone of 51 lakes within the NBW using a hand held piston coring sampler during the months between May through November from 2012–2013. The 51 lakes had surface areas of 3.4 to 420 ha, mean depths of 1.1 to 5.5 m, and covered a gradient of anthropogenic development in their watersheds. The surface layer of the intact core was re-suspended by stirring and the suspended material was removed by a large pipette. Samples were placed in clean plastic bottles, capped, placed on ice and transported to the laboratory and refrigerated until they were processed within 72 hours. Samples were stirred, poured through a 0.5 mm Nytex® screen (to remove large plant material, leaves, twigs, gravel, etc.) and collected in 55 ml centrifuge tubes. Samples were shaken and centrifuged at 1000g for five minutes, supernatant was poured off and a spatula was used to take samples from the top ~1.5cm of the material in the bottom of the tube. Samples were then dried in an oven at 40°C for > 3 days, ground with mortar and pestle to a fine powder and aliquots were placed into tin sample boats and weighed to 0.01mg on a microbalance. Stable isotope ratios of these BOM samples were determined using a continuous flow isotope ratio mass spectrometer (Isoprime 100 Mass Spectrometer, Elementar Americas, Mt. Laurel, NJ, USA) and

reported as per mil differences (‰) between samples and reference materials ($\delta^{15}N$ and $\delta^{13}C$). Atmospheric N_2 and Pee Dee Belemnite were reference standards for nitrogen and carbon stable isotopes, respectively [3, 15].

References for Supplementary Materials

1. Lin J.; Compton, J.E.; Leibowitz, S.G.; Mueller-Warrant, G.; Matthews, W.; Schoenholtz, S.H.; Evans, D.M. Seasonality of nitrogen balances in a Mediterranean climate watershed, Oregon, US, **2018** (in submission).
2. Evans D.M., S.H. Schoenholtz, P.J. Wigington Jr., S.M. Griffith, W.C. Floyd. Spatial and temporal patterns of dissolved nitrogen and phosphorus in surface waters of a multi-land use basin. *Environ. Moni. Assess.* **2014**, *186*,873–887.
3. Morrissey, C.A., Boldt, A., Mapstone, A., Newton, J., Ormerod, S.J. Stable isotopes as indicators of wastewater effects on the macroinvertebrates of urban rivers. *Hydrobiologia* **2013**, *700*, 231–244.
4. U.S. EPA, National Rivers and Streams Assessment 2013-2014: Field Operations Manual -Wadeable. EPA-841-B-12-009b. U.S. Environmental Protection Agency, Office of Water: Washington, DC, USA, 2013.
5. Kaufmann, P.R. *Physical Habitat Characterization in Environmental Monitoring and Assessment Program—Surface Waters Western Pilot Study: Field Operations Manual for Wadeable Streams*; Peck, D.V., Herlihy, A.T., Hill, B.H., Hughes, R.M., Kaufmann, P.R., Klemm, D.J., Lazorchak, J.M., McCormick, F.H., Peterson, S.A., Ringold, P.L., et al., Eds.; EPA/620/R-06/003; U.S. Environmental Protection Agency: Washington, DC, USA, 2006, pp. 111–174.
6. Kaufmann, P.R.; Levine, P.; Robison, E.G.; Seeliger, C.; Peck, D.V. *Quantifying Physical Habitat in Wadeable Streams*; EPA/620/R-99/003; U.S. Environmental Protection Agency: Washington, DC, USA, 1999.
7. Dunham, J.; Chandler, G.L.; Rieman, B.; Martin, D. *Measuring Stream Temperature with Digital Data Loggers: A User's Guide*; General Technical Report, RMRS-GTR-150WWW; U.S. Department of Agriculture, Forest Service, Rocky Mountain Research Station: Fort Collins, CO, USA, 2005.
8. R Core Team. 2017. R: A Language and Environment for Statistical Computing. R Foundation for Statistical Computing. Vienna, Austria. Available online: https://www.R-project.org (accessed on 1 May 2018).
9. Maheu, A.; Poff, N.L.; St-Hilaire, A. A classification of stream water temperature regimes in the conterminous USA. *River Res. Appl.* **2015**, *32*, 896–906.
10. Peck, D.V.; Herlihy, A.T.; Hill, B.H.; Hughes, R.M.; Kaufmann, P.R.; Klemm, D.J.; Lazorchak, J.M.; McCormick, F.H.; Peterson, S.A.; Ringold, P.L.; et al. (Eds.) *Environmental Monitoring and Assessment Program—Surface Waters Western Pilot Study: Field Operations Manual for Wadeable Streams*; EPA/620/R-06/003; U.S. Environmental Protection Agency: Washington, DC, USA, 2006.
11. Hughes, R.M.; McCormick, F.H. Aquatic Vertebrates. In *Environmental Monitoring and Assessment Program--Surface Waters: Western Pilot Study Field Operations Manual for Wadeable Streams*; Peck, D.V., Lazorchak, J.M., Klemm, D.J., Eds.; EPA/620/R-06/003; U.S. Environmental Protection Agency: Washington, DC, USA, 2006; pp. 225–250.
12. Whittier, T.R.; Hughes, R.M.; Stoddard, J.L.; Lomnicky, G.A.; Peck, D.V.; Herlihy, A.T. A structured approach for developing indices of biotic integrity: Three examples from streams and rivers in the western USA. *Trans. Am. Fish. Soc.* **2007**, *136*, 718–735.
13. Hicks, K.A.; Loomer, H.A.; Fuzzen, M.L.M.; Kleywegt, S.; Tetreault, G.R.; McMaster, M.E.; Servos, M.R. $\delta^{15}N$ tracks changes in the assimilation of sewage-derived nutrients into a riverine food web before and after major process alterations at two municipal wastewater treatment plants. *Ecol. Indic.* **2017**, *72*, 747–758.
14. Smucker, N.J.; Kuhn, A.; Charpentier, M.A.; Cruz-Quinones, C.J.; Elonen, C.M.; Whorley, S.B.; Jicha, T.M.; Serbst, J.R.; Hill, B.H.; Wehr, J.D. Quantifying Urban Watershed Stressor Gradients and Evaluating How Different Land Cover Datasets Affect Stream Management. *Environ. Manag.* **2016**, *57*,683–695.
15. Smucker, N.J.; Kuhn, A.; Cruz-Quinones, C.J.; Serbst, J.R.; Lake, J.L. Stable isotopes of algae and

macroinvertebrates in streams respond to watershed urbanization, inform management goals, and indicate food web relationships. *Ecol. Indic.* **2018**, *90*, 295–304.
16. Lake, J.L.; Serbst, J.R.; Kuhn, A.; Smucker, N.J.; Edwards, P.; Libby, A.; Charpentier, M.A.; Miller, K. Use of Stable Isotopes in Benthic Organic Material to Assess Watershed Impacts and Trophic Positions in Lakes. *Can. J. Fish. Aquat. Sci.* **2018,** under revision for publication.

© 2018 by the authors. Submitted for possible open access publication under the terms and conditions of the Creative Commons Attribution (CC BY) license (http://creativecommons.org/licenses/by/4.0/).
